# Discovery of synthetic G-quadruplex DNA as SARS-CoV-2 helicase inhibitor with antiviral, anti-inflammatory and antioxidative properties

**DOI:** 10.1038/s41420-026-03006-0

**Published:** 2026-03-18

**Authors:** Denisa Bojkova, Katja Steinhorst, Marco Bechtel, Nadja Zoeller, Monika Doll, Melanie Ott, Florian Rothweiler, Tamara Rothenburger, Kristoffer Riecken, Boris Fehse, Joshua D. Kandler, Ruth Olmer, Lucia Alcober-Boquet, Martin Michaelis, Jindrich Cinatl, Stefan Kippenberger

**Affiliations:** 1 Institute for Medical Virology, Johann Wolfgang Goethe University, Frankfurt/Main, Germany; 2 Department of Dermatology, Venereology and Allergy, Johann Wolfgang Goethe University, Frankfurt/Main, Germany; 3 Interdisciplinary Laboratory for Pediatric Tumor and Virus Research, Dr. Petra Joh Research Institute, Frankfurt/Main, Germany; 4 Department of Stem Cell Transplantation, Research Department Cell and Gene Therapy, University Medical Center Hamburg-Eppendorf, Hamburg, Germany; 5 German Center for Infection Disease (DZIF), Partner Site Hamburg-Lübeck-Borstel-Riems, Hamburg, Germany; 6 Leibniz Research Laboratories for Biotechnology and Artificial Organs (LEBAO), Department of Cardiothoracic, Transplantation and Vascular Surgery (HTTG), REBIRTH-Research Center for Translational Regenerative Medicine, Biomedical Research in Endstage and Obstructive Lung Disease Hannover (BREATH), German Center for Lung Research (DZL), Hannover Medical School, Hannover, Germany; 7 Medical Clinic 1, Johann Wolfgang Goethe University, Frankfurt/Main, Germany; 8 School of Natural Sciences, University of Kent, Canterbury, UK

**Keywords:** Viral infection, Target identification, Experimental models of disease, Chronic inflammation

## Abstract

SARS-CoV-2 RNA contains guanine-rich sequences that form secondary structures known as G quadruplexes (G4s). The SARS-CoV-2 nonstructural protein (NSP13) resolves G4s due to its helicase and ATPase activity, a process essential for viral replication. Here, we tested the effects of synthetic G4s on SARS-CoV-2 replication. In agreement, a synthetic G4 DNA 20 mer, consisting exclusively of guanines linked by a phosphorothioate backbone (designated GQ20-PTO), inhibited the replication of various SARS-CoV-2 variants in human lung cell cultures. Mechanistically, GQ20-PTO bound to NSP13 and inhibited its helicase and ATPase activity. Independent of its antiviral effects, GQ20-PTO additionally suppressed IFNβ and IL-6 (but not TNFα) signaling and the formation of reactive oxygen species, processes known to contribute to hyperinflammation in severe COVID-19. Hence, G4 quadruplexes like GQ20-PTO represent a novel class of DNA-based compounds for COVID-19 treatment with the potential to interfere with both SARS-CoV-2 replication and the uncontrolled inflammation associated with life-threatening COVID-19.

## Introduction

Since 2002, three novel coronaviruses (SARS-CoV, MERS-CoV, SARS-CoV-2) have caused zoonotic outbreaks in humans, with SARS-CoV-2 causing the coronavirus disease 2019 (COVID-19) pandemic [1–3]. The therapeutic arsenal for the treatment of SARS-CoV-2 infections is limited. Approved drugs include the RNA-dependent RNA polymerase inhibitor remdesivir, the 3C-like protease inhibitor nirmatrelvir, and molnupiravir that induces error catastrophe in the viral genome [1, 4]. In addition, corticosteroids, Janus kinase-1/2 (JAK1/2), and IL-6 receptor antagonists are clinically used to treat the hyperinflammation (‘cytokine storm’) associated with life-threatening COVID-19 [4–6].

Due to its high sequence conservation and crucial role in SARS-CoV-2 replication the SARS-CoV-2 nonstructural protein 13 (NSP13) is considered as an additional target for the development of antiviral drugs [7, 8]. NSP13 possesses both helicase and ATPase activity and unwinds double-stranded RNA or DNA in a 5’ to 3’ direction [7, 9]. During virus replication, the unwinding of double-stranded RNA dissociates RNA secondary structures such as G-quadruplexes (G4s). Furthermore, NSP13 interacts with the RNA-dependent polymerase NSP12 as part of the replication-transcription complex (NSP7/NSP8/NSP12), which is responsible for the replication and transcription of the SARS-CoV-2 genome [10]. Recently, several potential inhibitors of NSP13, which suppress its helicase and/or ATPase activity, have been identified [8, 11–13].

G4s are built by guanine-rich nucleic acids forming intramolecular planar arrangements through Hoogsteen hydrogen bonds, which have been detected in the genomes of mammals (including humans), bacteria, and viruses (including SARS-CoV-2) [14, 15]. It is proposed that G4s stabilize the genome by forming “knots” that work as stable barriers to the progression of molecular motors tracking on the genome [16]. Moreover, G4s are thought to protect viral RNA from degradation by host cell nucleases [14]. The importance of G4 is documented by the observation that synthetic compounds stabilizing G4 exert antiviral activities [17–19].

We hypothesized that synthetic G4 DNA may interact with NSP13, prevent its binding to the G4s in the SARS-CoV-2 genome, and in turn the unwinding of double-stranded RNA and SARS-CoV-2 replication. Indeed, a synthetic G4 molecule consisting exclusively of guanines linked by a phosphorothioate (PTO) backbone, designated GQ20-PTO, was identified as a potent antiviral agent. GQ20-PTO binds to NSP13, inhibiting its helicase and ATPase activities, and strongly suppresses viral replication. In a previous study, we showed that the same synthetic G4 used here inhibits IFN-γ signaling in melanoma cells [20]. Moreover, PTO DNA oligonucleotides may display antioxidative properties [21]. This prompted us to investigate also the anti-inflammatory and antioxidative effect of GQ20-PTO in the context of SARS-CoV-2 infection. In agreement, GQ20-PTO also inhibited interferon and IL-6 signaling in SARS-CoV-2-infected cells by mechanisms independent of those involved in virus replication inhibition. Hence, GQ20-PTO may interfere with the COVID-19 disease course, both by antiviral and immunomodulatory effects.

## Results

### GQ20-PTO inhibits infection of SARS-CoV-2

**Fig. 1:**
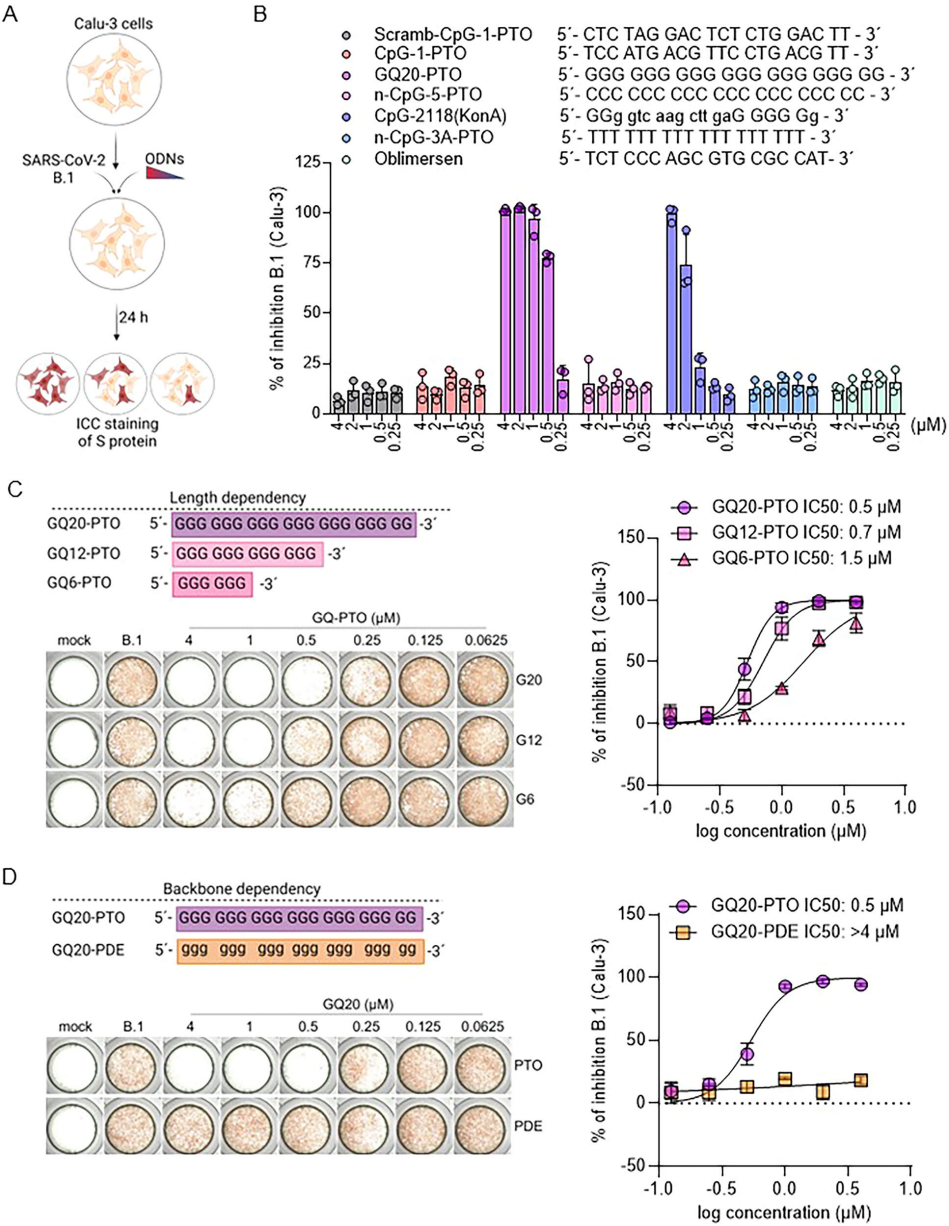
GQ20-PTO inhibits replication of SARS-CoV-2 in Calu-3 cells. **A** Schematic display of the experimental set-up. **B** Quantitative inhibition of SARS-CoV-2 replication in Calu-3 cells by different ODNs, concentrations are indicated; capitals denote phosphorothioates and lowercase letters denote phosphodiesters. **C** Different lengths of mutants of GQ20-PTO are depicted. Representative images illustrate immunohistochemical staining of SARS-CoV-2 S protein in treated Calu-3 cells. On the right, the dose-dependent inhibition of SARS-CoV-2 replication with calculated IC_50_ values. **D** Comparison of phosphorothioate (PTO) and phosphodiester (PDE) backbone on GQ20 anti-SARS-CoV-2 efficacy. Representative images illustrate immunohistochemical staining of SARS-CoV-2 S protein in treated Calu-3 cells. On the right, a graphical representation of the backbone-dependent inhibition on SARS-CoV-2 replication.

Calu-3 cells were infected with SARS-CoV-2 (B.1) and treated with different oligonucleotides (ODNs) as displayed in Fig. 1A. Immunofluorescence staining of S antigen was used to determine the viral infection. Among tested ODNs, GQ20-PTO, a 20-nucleotide ODN composed solely of guanosines connected by a phosphorothioate backbone, exhibited the most potent inhibition of SARS-CoV-2 infection at concentrations between 0.25 and 4 µM. (Fig. 1B). The second most potent ODN was CpG-2118(KonA), which consists of 12 guanosines within a 20-nucleotide sequence. This ODN was originally designed as a control for a CpG-containing ODN (ODN 1585) in which the CG sequence was inverted to GC [22]. All other ODNs lacked an inhibitory effect on SARS-CoV-2 infection.

### GQ20-PTO-mediated inhibition of the virus depends on molecule length and backbone composition

To test whether the inhibitory effect on SARS-CoV-2 replication depends on the molecule length, different lengths of mutants of GQ20-PTO were tested (Fig. 1C). It was found that the inhibitory effect decreased with shorter ODN. The IC_50_ for GQ20-PTO was 0.5 µM, while GQ12-PTO, with 12 nucleotides, had an IC_50_ of 0.7 µM and GQ6-PTO, with 6 nucleotides, had an IC_50_ at 1.5 µM. Next, the effect of the ODN backbone was tested. Here we compared GQ20-PTO, which has a total phosphorothioate backbone with GQ20-PDE, with the same sequence but a phosphodiester backbone (Fig. 1D). GQ20-PTO showed the expected strong inhibition of SARS-CoV-2 (IC_50_: 0.5 µM), while GQ20-PDE exerted no antiviral effect (IC_50_: >4 µM). Importantly, both GQ20-PTO and GQ20-PDE were non-toxic in Calu-3 cells and normal human fibroblasts (CC_50_: >8 µM) (Supplementary Fig. S1).

### GQ20-PTO forms G-quadruplexes (G4) and is efficiently taken up by cells

**Fig. 2:**
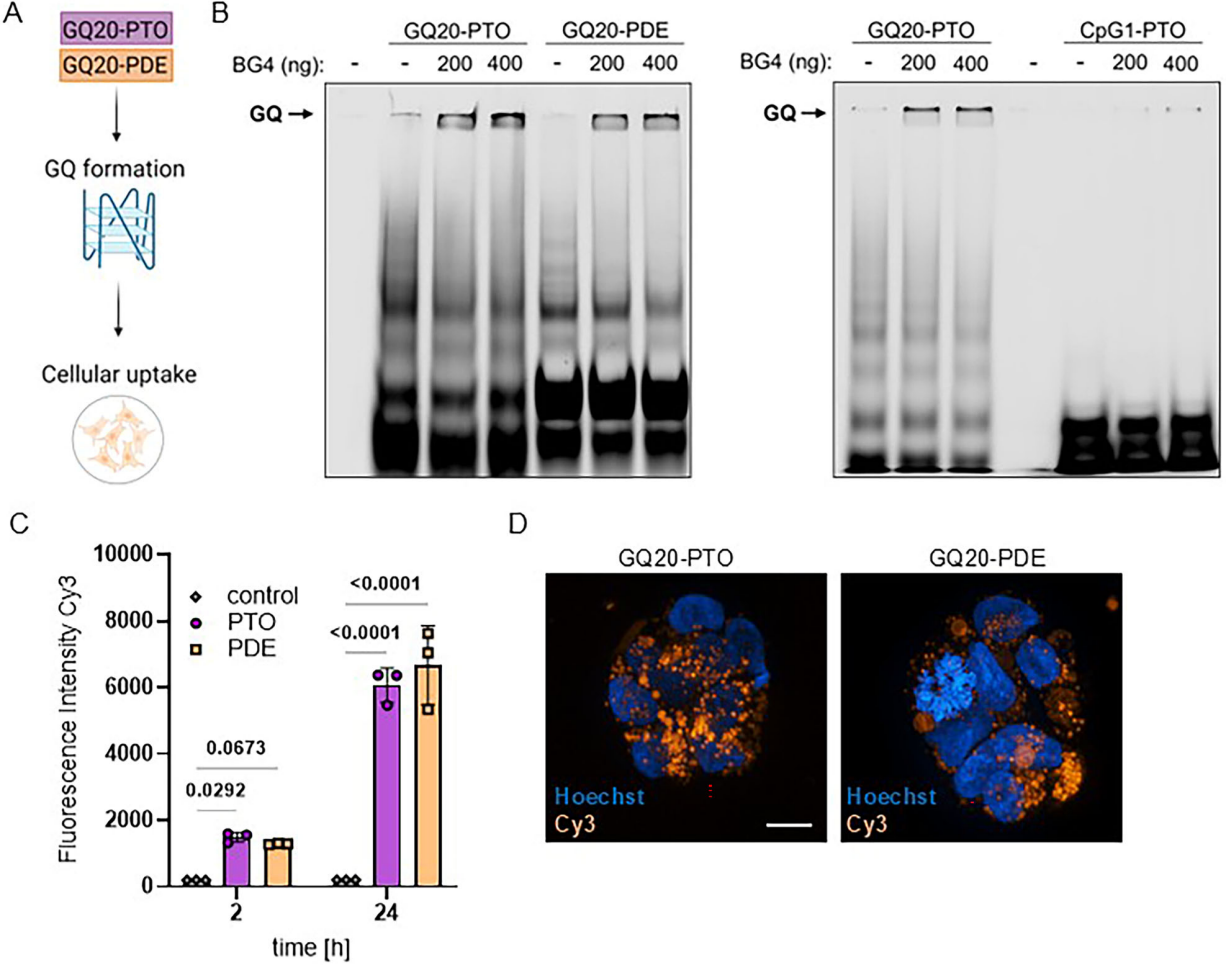
GQ20 forms quadruplexes (G4) and is taken up by cells. **A** Scheme of experimental endpoints. **B** Binding of Cy3-labeled GQ20-PTO, GQ20-PDE, and CpG-1-PTO to BG4, an antibody recognizing G4, demonstrated by in vitro gel shift assays. The complex between ODN and BG4 is indicated (GQ). **C** Quantitative uptake of 1 µM Cy3-labeled GQ20-PTO and GQ20-PDE into Calu-3 cells. Graph displays the relative Cy3/Hoechst 33324 intensity; *P* values are indicated. **D** confocal images taken after 24 h. scale bar: 10 µm.

From the molecular characteristics of GQ20-PTO it seems likely that the antiviral effect depends on secondary structures known as G-quadruplexes (G4) (schematically depicted in Fig. 2A). Therefore, we investigated if there is any difference between GQ20-PTO and GQ20-PDE regarding the presence of G4 structures. Figure 2B shows the formation of G4 by using Cy3-labeled ODNs in combination with BG4, a G4-binding antibody, as described by Moruno-Manchon et al. [23]. GQ20-PTO and GQ20-PDE show a widespread pattern of secondary structures which were mostly absent in the control ODN, CpG-1-PTO. When ODN were mixed with 200 ng or 400 ng BG4 before gel loading, GQ20-PTO and GQ20-PDE were shifted to the upper section of the gel. Moreover, the presence of BG4 weakened the signals derived from secondary structures, indicating adsorption of G4 to BG4. No gel-shift was found for CpG-1-PTO, confirming the G4 specificity of BG4. To study the cellular uptake, Calu-3 cells were incubated with 1 µM Cy3-labeled GQ20-PTO or GQ20-PDE for 2 h and 24 h. Quantitative analysis showed no significant difference in cellular fluorescence between both molecules (Fig. 2C). Confocal images taken after 24 h showed intracellular accumulation of Cy3 fluorescence in distinct spots within the cytoplasm (Fig. 2D). A 3D animation composed of multiple z-stack images displays the cellular distribution (Supplementary Fig. S2).

### GQ20-PTO inhibits different SARS-CoV-2 variants – inhibition of virus entry and post-entry effects

**Fig. 3:**
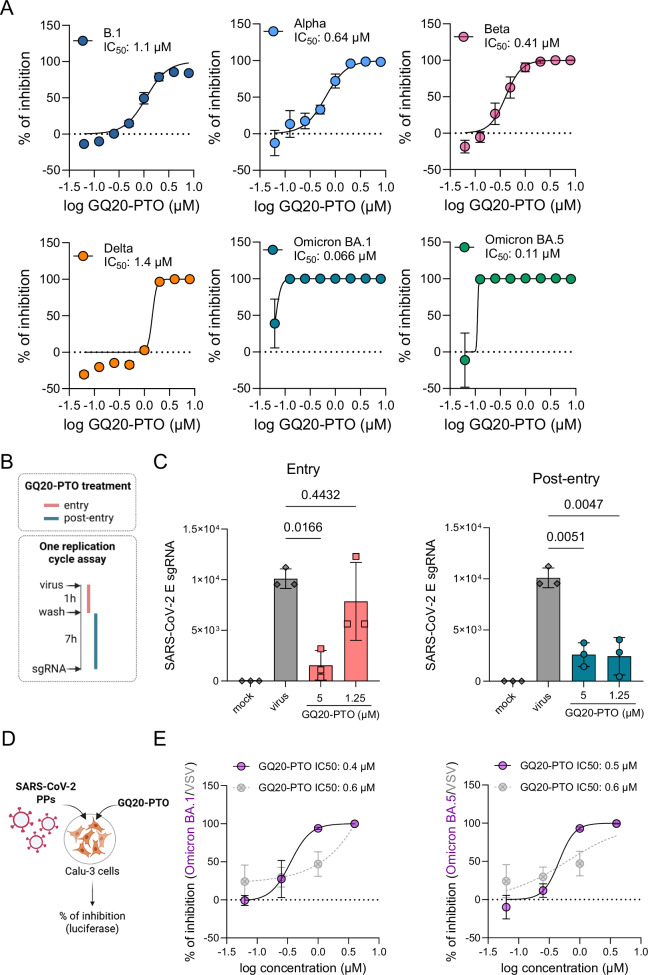
GQ20-PTO inhibits different SARS-CoV-2 variants, inhibits virus entry and replication. **A** Dose-dependent effect of GQ20-PTO on infection of different SARS-CoV-2 variants. **B** Schematic display of the experimental set-up to study entry and post-entry effects. **C** Summarized data of E gene RNA expression discriminating between entry and post-entry effects of GQ20-PTO. **D** Schematic depictions of the experimental set-up of pseudovirus assay based on lentiviral vectors. **E** Graph displays dose-dependent effects of GQ20-PTO on Omicron BA.1/BA.5 S protein internalization of pseudotyped lentiviral vectors. Lentiviral vectors pseudotyped with vesicular stomatitis virus G protein (VSV-G)(in gray) were used as a specificity control. IC_50_ values are indicated.

The inhibitory effect of GQ20-PTO was evaluated on a set of SARS-CoV-2 variants including B.1, Alpha, Beta, Delta, Omicron BA.1 and Omicron BA.5 (Fig. 3A). GQ20-PTO effectively inhibited infection across all tested virus variants, though with quantitative differences. Both Omicron strains displayed stronger suppression of their replication (IC_50_: Omicron BA.1, 0.066 µM; Omicron BA.5, 0.11 µM) in comparison to previous variants (IC_50_: B.1, 1.1 µM; Alpha, 0.64 µM; Beta, 0.41 µM; Delta, 1.4 µM).

Next, we conducted time-of-addition experiments to distinguish between virus entry and post-entry effects of GQ20-PTO (Fig. 3B). As read-out, we assessed the production of subgenomic RNA (sgRNA) of the E gene 8 hours post infection, corresponding to one replication cycle. The data on SARS-CoV-2 E sgRNA expression indicated that both virus entry and post-entry effects are inhibited at 5 µM GQ20-PTO, whereas only post-entry effects were inhibited at 1.25 µM (Fig. 3C). To further investigate the effect on virus entry, we utilized pseudoviral particles (PPs) based on lentiviral vectors pseudotyped with the SARS-CoV-2 S protein and expressing a luciferase reporter [24] (Fig. 3D). GQ20-PTO inhibited uptake of both BA.1 (IC_50_: 0.4 µM) and BA.5 (IC_50_: 0.5 µM) PPs (Fig. 3E). Notably, GQ20-PTO also partially inhibited the entry of lentiviral vectors pseudotyped with VSV-G, which served as the control PP (IC_50_: 0.6 µM). These findings suggest that GQ20-PTO displaces antiviral activity both by non-specific effects on virus entry and by targeting post-entry events. The subsequent experiments were designed to further characterize these post-entry effects.

### GQ20-PTO binds to SARS-CoV-2 NSP13, inhibiting helicase and ATPase activity

**Fig. 4:**
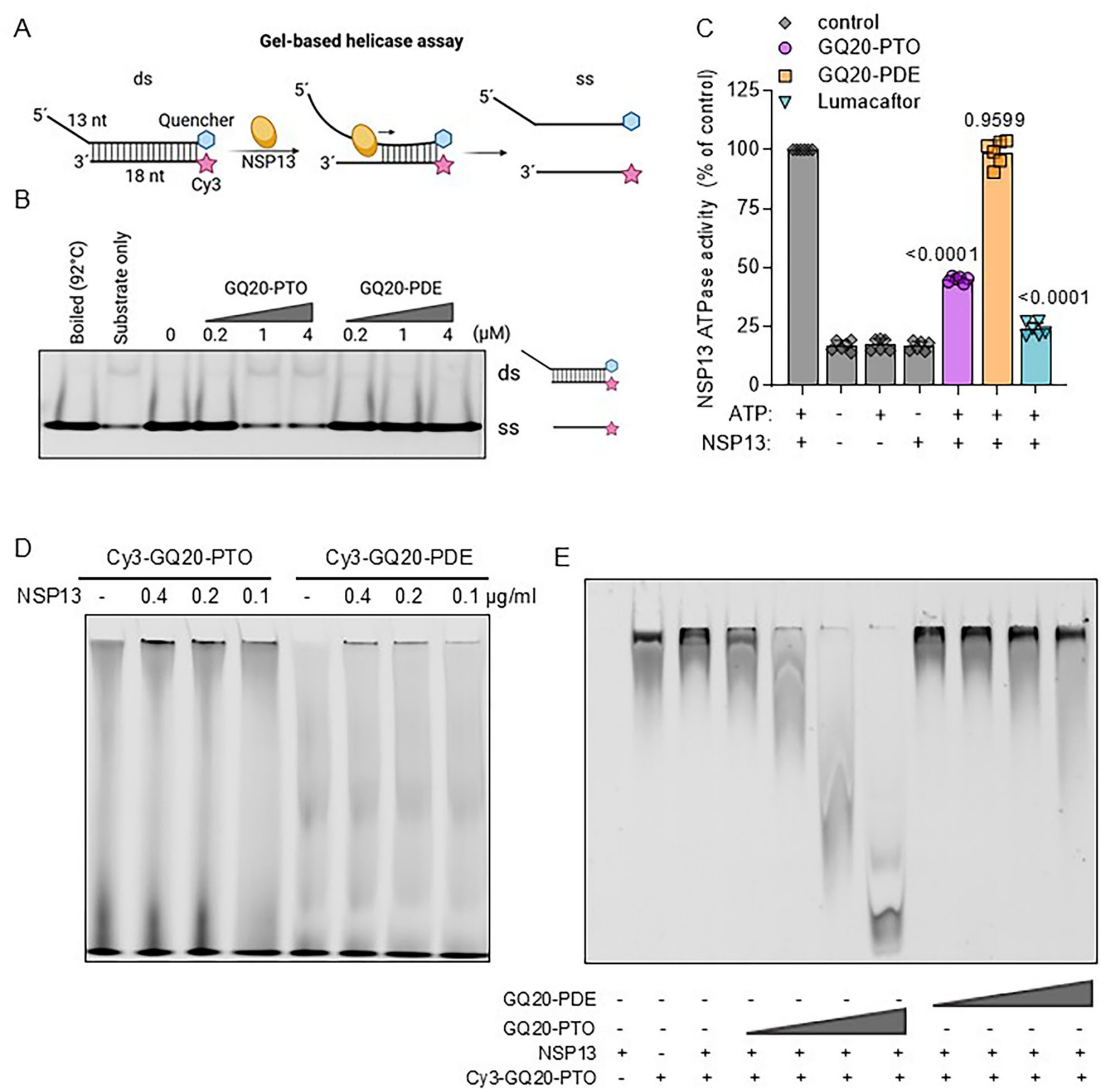
GQ20-PTO inhibits SARS-CoV-2 NSP13 helicase and ATPase activity. **A** Scheme of the NSP13 helicase assay. The separation of DNA strands by nsp13 produces fluorescence corresponding to NSP13 activity. **B** The helicase activity of NSP13 is displayed in a gel-based helicase assay; the positions for double-stranded DNA (ds) and separated strands (ss) are indicated. The decrease of single-stranded DNA by GQ20-PTO indicates inhibition of NSP13. **C** ATPase activity of the SARS-CoV-2 NSP13 after GQ20-PTO and GQ20-PDE treatment. 1 mM Lumacaftor served as positive control to display maximum inhibition. Each column represents the mean of at least six independent experiments. Statistical analysis was performed in relation to controls (NSP13, ATP) using Student´s *T* test with *P* values indicated in the graph. **D** Binding of 60 nM Cy3-labeled GQ20-PTO or GQ20-PDE to different amounts of NSP13 (9.8, 4.9, 2.4 µg/mL). **E** Binding of 60 nM Cy3-labeled GQ20-PTO to 9.8 µg/mL NSP13 is displaced by increasing amounts of unlabeled GQ20-PTO (60, 240, 960, 3840 nM), increasing amounts of unlabeled GQ20-PDE did not.

It has been reported that viral helicases including NSP13 from SARS-CoV-2 interact with G4 structures [25]. To test whether GQ20-PTO interferes with virus helicase, we examined four aspects of NSP13 in the context of GQ20-PTO: (a) helicase activity, (b) ATPase activity, (c) binding, and (d) stability. To assess the NSP13 helicase activity, we evaluated the separation of a double-stranded DNA fragment as illustrated in Fig. 4A. In a gel-based assay, separation of the double-stranded DNA fragment is indicated by the appearance of a fluorescent single strand, where the fluorescent signal is initially quenched. Complete separation was achieved by melting the double-stranded DNA fragment at 92 °C, which served as a positive control. In the presence of GQ20-PTO a concentration-dependent inhibition of NSP13 helicase activity was observed indicated by the decrease of fluorescence (Fig. 4B). Notably, no inhibition was found for GQ20-PDE in the examined concentration range. Since all helicases possess an intrinsic ATPase activity crucial for energetically stable DNA unwinding, we further investigated the effect of GQ20-PTO on NSP13 ATPase activity (Fig. 4C). The maximum ATPase activity in the presence of NSP13 and ATP was set to 100%. Treatment with 1 mM lumacaftor, a previously described inhibitor of SARS-CoV-2 NSP13 [8], reduced ATPase activity to 24%. Likewise, a treatment with 4 µM GQ20-PTO inhibited ATPase activity to 44%. In contrast, treatment with 4 µM GQ20-PDE has no inhibitory effect. In summary we found that GQ20-PTO shows inhibition of both helicase and ATPase activity in SARS-CoV-2 NSP13.

Next, we applied an in vitro gel shift assay to evaluate the binding of NSP13 to a Cy3-coupled GQ20-PTO. We observed a dose-dependent shift under GQ20-PTO treatment, suggesting its binding to NSP13 (Fig. 4D). Interestingly, the corresponding PDE molecule (Cy3-GQ20-PDE) also bound to NSP13, although distinctively weaker. We hypothesized that NSP13 inhibition depends on the binding strength of both molecules. Therefore, competitor experiments were performed (Fig. 4E). It was found that the binding of Cy3-GQ20-PTO to NSP13 decreased by the addition of unlabeled GQ20-PTO in a concentration-dependent manner. Notably, the addition of increasing concentrations of GQ20-PDE did not displace Cy3-GQ20-PTO from NSP13. This indicates that the PTO modification in GQ20 enables a stronger binding to NSP13 in comparison to PDE.

Lastly, we evaluated the effect of GQ20-PTO on NSP13 ectopically overexpressed in HEK293T cells. The addition of GQ20-PTO did not show any effect on NSP13 protein stability (Supplementary Fig. S3A). Thermal stability assay displayed only marginal changes of NSP13 melting points between untreated (V50: 54.7 °C) and GQ20-PTO-treated NSP13 (V50: 54.2 °C) (Supplementary Fig. S3B). Taken together, these findings indicate that GQ20-PTO inhibits NSP13 by binding and interfering with the enzymatic activity without a significant effect on NSP13 stability.

### GQ20-PTO inhibits SARS-CoV-2 replication in human bronchial epithelium

**Fig. 5:**
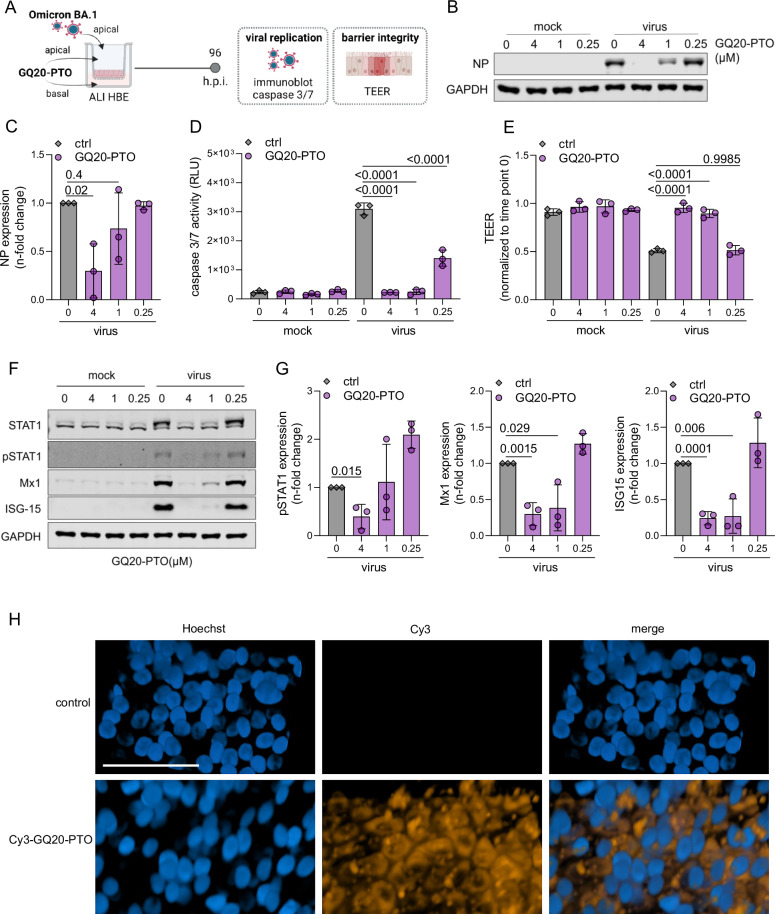
GQ20-PTO inhibits virus replication in ALI cultures of primary human bronchial epithelial (HBE) cells. **A** Schematic display of the experimental set-up. **B** Immunoblot analysis of viral nucleocapsid protein (NP) in the presence of GQ20-PTO. **C** Densitometric analysis of NP. Signals were normalized to GAPDH and related to the virus control (ctrl). Statistical analysis was performed in relation to virus control using one-way ANOVA with *P* values indicated in the graph. **D** Quantitative levels of virus-induced caspase 3/7 activation in the presence of GQ20-PTO. *P* values of one-way ANOVA are indicated. **E** Quantitative measurement of the transepithelial electrical resistance (TEER) after virus infection in the presence of GQ20-PTO. *P* values of one-way ANOVA are indicated in the graph. **F** Immunoblot analysis of interferon (IFN) signaling of infected and treated ALI cultures. **G** Densitometric analysis of pSTAT, Mx1 and ISG15 shown in (**F**). *P* values of one-way ANOVA are indicated in the graph. **H** Representative images of HBE ALI cultures treated with (1 µM) Cy3-labeled GQ20-PTO (applied apically and to the basal medium) for 24 h; Cy3 in orange, Hoechst 33324 in blue. Scale bar: 50 µm.

The antiviral efficiency of GQ20-PTO was further evaluated using 3D models of the human pseudostratified bronchial epithelial barrier, cultured at the air–liquid interface (HBE ALI), as displayed in Fig. 5A. GQ20-PTO dose-dependently suppressed SARS-CoV-2 replication as evidenced by the reduced expression of nucleocapsid protein (NP) (Fig. 5B, C). Moreover, all tested GQ20-PTO concentrations (0.25, 1, 4 µM) significantly mitigated virus-induced apoptosis in ALI HBE cultures as displayed by reduced caspase 3/7 activity (Fig. 5D). In addition, GQ20-PTO treatment preserved the barrier integrity as assessed by quantitative measurement of the transepithelial electrical resistance (TEER), in infected ALI HBE cultures (Fig. 5E). These findings suggest that GQ20-PTO may protect bronchial epithelium beyond suppression of virus replication.

Previous studies have linked tissue damage during SARS-CoV-2 infection to an excessive interferon (IFN) response in airway epithelium [26]. Therefore, we investigated the effects of GQ20-PTO on the IFN signaling pathway in ALI HBE cultures during SARS-CoV-2 infection (Fig. 5F). Infection with SARS-CoV-2 alone led to the upregulation of STAT1, pSTAT1, Mx1, and ISG15 (Fig. 5F). In contrast, GQ20-PTO treatment suppressed their expression (Fig. 5F, G). Notably, GQ20-PTO inhibited the expression of both Mx1 and ISG15 even at concentrations that did not significantly reduce virus replication, suggesting potential virus-independent effects of GQ20-PTO on the IFN signaling pathway. Confocal imaging of HBE ALI cultures treated with 1 µM Cy3-labeled GQ20-PTO for 24 hours demonstrated evenly distributed intracellular fluorescence. Counterstaining with Hoechst 33324 indicated that GQ20-PTO localizes in the cytoplasm but not in nuclei (Fig. 5H).

### GQ20-PTO suppresses inflammation and ROS

**Fig. 6:**
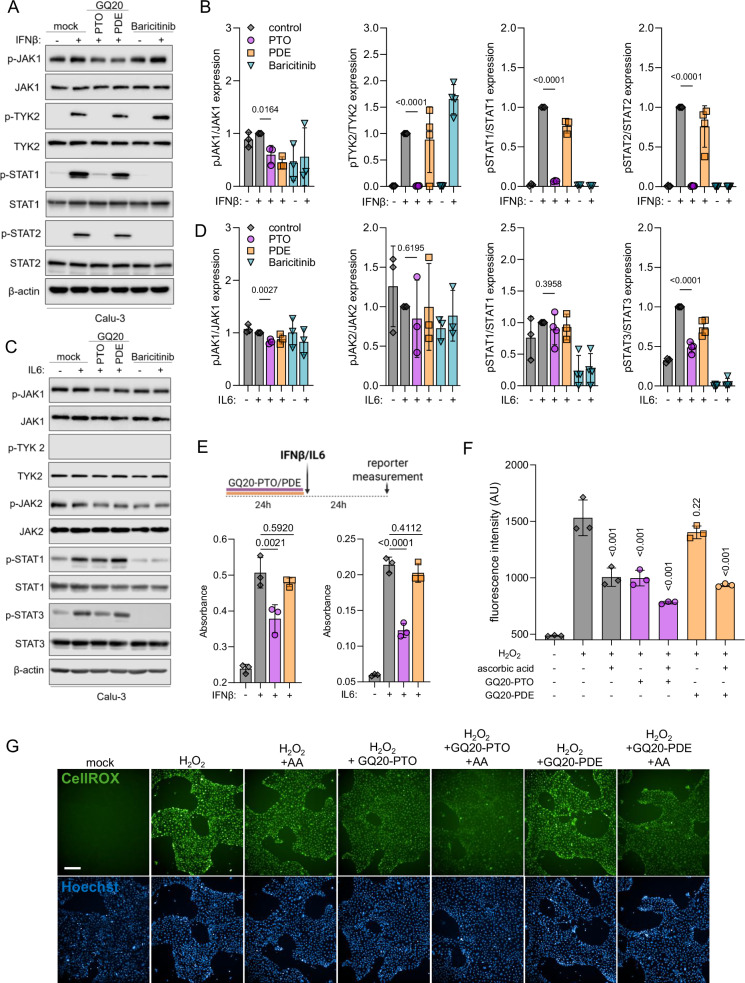
GQ20-PTO suppresses inflammatory signaling molecules and diminishes oxidative stress. Calu-3 cells were treated with **A** 20 ng/mL IFNβ or **C** 20 ng/mL IL-6 in the presence of 4 µM GQ20-PTO, GQ20-PDE or 1 µM baricitinib for 10 min and then analyzed by western blotting using the indicated antibodies. Densitometric analysis of western blots is shown in (**B**, **D**). **E** Schematic representation of the experimental setup showing HEK293 reporter cells engineered to express human IFNAR1/IFNAR2 or IL-6 receptors. Left: Stimulation of IFNAR1/R2 reporter cells by IFNβ (positive control) in the presence of 4 µM GQ20-PTO or GQ20-PDE. Right: Stimulation of IL-6 reporter cells by IL-6 (positive control) in the presence of 4 µM GQ20-PTO or GQ20-PDE. **F** Quantitative results of ROS formation in 16HBE14o cells after addition of 1 mM H_2_O_2_. Each bar represents the mean of three independent experiments. Significance test (one-way ANOVA) was performed in comparison to the positive control; *P* values are indicated. **G** Representative images of CellROX and Hoechst 33324 staining. Scale bar: 200 µm.

To investigate virus-independent inflammation, Calu-3 cells were pre-treated with GQ20-PTO, GQ20-PDE, or baricitinib (JAK1/2 inhibitor) and then stimulated with either IFNβ or IL-6 for 10 min. Both cytokines are known to aggravate the course of COVID-19 by excessive and persistent inflammation [27, 28]. Immunoblot analysis of key signaling molecules in type I IFN and IL-6 pathways is shown in Fig. 6A, C, respectively; quantitative results are given in Fig. 6B, D.

GQ20-PTO treatment completely suppressed phosphorylation of TYK2, STAT1 and STAT2 after IFNβ stimulation (Fig. 6A, B) and STAT3 after IL6 treatment (Fig. 6C, D), whereas GQ20-PDE had no effect. Of note, JAK1 is constitutively activated in Calu-3 cells with IFNβ and IL-6, causing no further increase in phosphorylation. Statistical analysis showed a small but significant suppression of JAK1 activation by GQ20-PTO (Fig. 6B, D). IL-6 caused only for STAT3 a distinct activation in Calu-3; JAK2 and STAT1 were not activated (Fig. 6C, D).

Since the innate immune response seems to be deregulated in Calu-3 cells, we have investigated the anti-inflammatory effect of GQ20-PTO in a primary cell model using human pulmonary fibroblasts. In these cells, JAK1, TYK2, STAT1, and STAT2 were markedly phosphorylated in response to IFNβ treatment (Supplementary Fig. S4A). Pre-treatment with GQ20-PTO reversed the phosphorylation to basal levels. In contrast, IL-6 induced JAK1, STAT1, and STAT3 phosphorylation, whereas no TYK2 activation was observed, which might be a time-kinetic issue (Supplementary Fig. S4B). However, GQ20-PTO inhibited activation of JAK1, STAT1 and STAT3. To test if this is caused by some non-specific effect, additionally we evaluated the impact of GQ20-PTO on the TNFα pathway. GQ20-PTO showed no effect on TNFα-induced p38 phosphorylation (Supplementary Fig. S5A–C).

Additionally, we analyzed the inhibitory effect of GQ20-PTO on type I IFN and IL-6 signaling by using reporter cells expressing a colorimetric reporter gene for the IFNα/β and IL-6 signaling pathway. The experimental procedure is schematically depicted in Fig. 6E. Pre-treatment with 4 µM GQ20-PTO resulted in 76.9% reduction of IFNα/β-induced promoter activity (Fig. 6E, left panel) and 65% reduction of IL-6-induced promoter activity (Fig. 6E, right panel). Treatment with GQ20-PDE had no effect. These data indicate the immunosuppressive effect of GQ20-PTO on two key pathways involved in hyperinflammation observed in COVID-19.

Finally, GQ20 was examined for its antioxidative property. Bronchial cells (16HBE14o) were pre-treated with GQ20-PTO, GQ20-PDE, and/or ascorbic acid and then stressed with 1 mM H_2_O_2_. The formation of ROS was detected by using the CellROX assay (Fig. 6H). GQ20-PTO and ascorbic acid (positive control) suppressed the ROS induction to the same level, whereas GQ20-PDE showed no significant reduction of ROS. Interestingly, the combination of GQ20-PTO and ascorbic acid further suppressed ROS induction.

## Discussion

Our results demonstrate that a G4-forming oligonucleotide, GQ20-PTO, is a novel SARS-CoV-2 inhibitor able to prevent SARS-CoV-2 infection in a 3D organotypic culture of human bronchial epithelium. Our findings indicate that viral inhibition is modulated by both oligonucleotide length and backbone chemistry. Shorter sequences (12-mer and 6-mer) exhibit diminished antiviral activity. Furthermore, oligonucleotides containing a fully phosphorothioate-modified backbone demonstrate potent antiviral effects, whereas the corresponding sequences with a phosphodiester backbone were ineffective. Phosphorothioates are known to resist nuclease degradation and thereby increasing the molecule half-life in biological systems. Moreover, they display enhanced binding to proteins [29, 30]. Interestingly, others have found that AS1411, a G4-forming aptamer with a PDE backbone [31], which is tested as anticancer drug [32], showed antiviral activity against SARS-CoV-2 at a concentration of 10 µM [33]. The exact mode of the antiviral action of AS1411 remains unclear, but an interference with the nucleolin-mediated trafficking seems likely [34]. In our experiments, we show effective inhibition of SARS-CoV-2 only for AS1411-PTO (IC_50_: 0.45 µM), the original unmodified AS1411 has no antiviral effect at concentrations up to 4 µM (Supplementary Fig. S6), confirming the increased antiviral effects of PTOs.

Although GQ20-PTO, at high micromolar concentrations, may display non-specific antiviral effects by interfering with virus uptake, we also observed antiviral activity at submicromolar concentrations after virus uptake in cells infected with different SARS-CoV-2 variants. Mechanistically, our experiments provide evidence that the SARS-CoV-2 helicase, NSP13, is the target for the GQ20-PTO-mediated viral inhibition. Both the helicase and ATPase activities of NSP13 are markedly decreased by GQ20-PTO. The genome of SARS-CoV-2 is rich in G4-forming sequences located in the open reading frames of ORF1 ab, spike (S), ORF3a, membrane (M) and nucleocapsid (N) genes which were shown to interact with NSP13 [25]. Therefore, it seems likely that artificial G4s serve as a fake substrate inhibiting NSP13 to unfold the virus genome. Notably, Shum and Tanner described that only non-G4 aptamers were able to inhibit SARS-CoV helicase unwinding activity and even stimulating ATPase activity [35]. This finding is surprising as NSP13 helicase shares the highest sequence conservation across the CoV family with a 99.8% sequence identity between SARS-CoV-2 and SARS-CoV with only one single residue difference [8]. A possible explanation for this discrepancy may be found in sequence specificity and backbone modification (only phosphodiester backbones were tested by Shum and Tanner [35]). Our data strongly indicate that the phosphorothioate backbone in GQ20-PTO is crucial for its effectiveness, since GQ20-PDE does not inhibit either unwinding or ATPase activity of NSP13. Vice versa, the relevance of the sequence is supported by experiments with CpG-1-PTO, a non-G4-forming 20-mer with a phosphorothioate backbone, which showed no antiviral activity and did not affect the ATPase function of NSP13 either. The importance of the backbone is supported by data showing that G4-forming RNA ODN with phosphorothioate backbone inhibits SARS-CoV-2 replication [36].

Previously, we have shown that GQ20-PTO inhibits IFNγ-induced JAK/STAT-signaling in melanoma cells and thereby suppresses the expression of PD-L1, which may support an immunologic anti-tumor response [20]. Remarkably, GQ20-PTO also suppresses key signaling pathways involved in virus-induced inflammation, the type I IFN and IL-6 signaling pathways, but not TNFα signaling. Both of these pathways were identified in a cohort of severely ill COVID-19 patients as drivers of the disease, associated with a prolonged and excessive deliberation of inflammatory cytokines triggering *inter alia* lung tissue damage [27, 37, 38]. This is highlighted by clinical studies showing that targeting these pathways has an effect on the course of the disease [39, 40]. However, immunosuppressive therapies can increase the risk for opportunistic infections, demanding a cautious application [41, 42].

Oxidative stress plays a key role in SARS-CoV-2-induced clinical conditions [43]. Particularly in long COVID, a chronic condition associated with SARS-CoV-2 infection, oxidative stress or redox imbalance has been identified as a contributing factor [44, 45]. Notably, we demonstrate that GQ20-PTO offers antioxidative properties by reducing the amount of ROS, a finding that is in line with previous studies showing the relevance of PTOs in this context [21, 46, 47].

In sum, our findings demonstrate a G4-forming ODN, GQ20-PTO, as an inhibitor of SARS-CoV-2 virus replication. In addition, this compound interferes with pro-inflammatory signaling cascades. Severe courses of COVID-19 associated with hyper-inflammatory conditions such as multisystem inflammatory syndrome in children (MIS-C) [48] and long COVID may benefit from treatment with GQ20-PTO. Unlike immunosuppression, which can be detrimental in acute viral infections, GQ20-PTO may be used as an antiviral *and* anti-inflammatory compound effective against a broad spectrum of SARS-CoV-2 variants. Although there is decades of experience with PTOs regarding stability and toxicity [29, 49], further studies are required prior to the clinical application of GQ20-PTO.

## Materials and methods

### Oligonucleotides (ODN)

ODN were synthesized and purified (HPLC) by BioSpring GmbH (Frankfurt/Main, Germany), reconstituted in water, and stored at –20 °C. The endotoxin content was <250 EU/g. ODN was given to the cells at the indicated concentration without DNA complexing reagents.

### Cell culture

Calu-3 cells (ATCC), human lung adenocarcinoma-derived cell line, 16HBE14o (Sigma-Aldrich), human epithelial bronchial cell line were maintained in MEM (Minimal Essential Medium) containing 10% (v/v) fetal bovine serum, 100 IU penicillin, 100 µg/ml streptomycin, and 2% (v/v) L-glutamine (Sigma-Aldrich, Taufkirchen, Germany). Human pulmonary fibroblasts (HPF, ScienCell, Carlsbad, USA) were propagated in fibroblast medium (ScienCell). Primary human bronchial epithelial (HBE) cells were isolated from lung explant tissue of a patient with lung emphysema as described previously [50, 51]. The use of tissue was approved by the ethics committee of the Hannover Medical School (MHH, Hannover, Germany, number 2923–2015) and complied with the Code of Ethics of the World Medical Association. For differentiation into air–liquid interface (ALI) cultures, the cells were thawed and passaged once in PneumaCult-Ex Medium (StemCell technologies, Cologne, Germany) and then seeded on transwell inserts (12-well plate, Sarstedt, Nümbrecht, Germany) at 4 × 10^4^ cells/insert. After reaching confluence, medium on the apical side of the transwell was removed, and medium in the basal chamber was replaced with PneumaCult ALI Maintenance Medium StemCell Technologies) including antibiotic/antimycotic solution (Sigma-Aldrich) and MycoZap Plus PR (Lonza, Cologne, Germany). Criteria for successful differentiation were the development of ciliary movement, an increase in transepithelial electric resistance (TEER, device, manufacturer, country), and mucus production.

### Virus preparation

Virus preparation was performed as described [50]. Briefly, SARS-CoV-2 variants were isolated using the human colon carcinoma cell line Caco-2. SARS-CoV-2 stocks used in the experiments had undergone a maximum of three passages on Caco-2 cells and were stored at −80 °C. Virus titers were determined as TCID_50_/mL in confluent cells in 96-well microtiter plates.

### Antiviral assay

Confluent layers of Calu-3 cells in 96-well plates were infected with SARS-CoV-2 at a multiplicity of infection (MOI) of 0.01. Virus was added together with the oligonucleotides at the same time and incubated in MEM supplemented with 1% FBS. The antiviral effects were assessed after 2 days either by immunohistochemical or immunofluorescent labeling of a virus-specific antigen using antibodies against SARS-CoV-2 S (1:1500, Sino Biological, Eschborn, Germany). The quantitative detection was performed using the Bioreader 7000 -F-Z-I micro (Bio-Sys, Karben, Germany).

### Immunofluorescent/immunohistochemical detection of SARS-CoV-2

Cells were fixed with methanol/acetone (60/40) and blocked with 2% BSA and 5% goat serum. Staining was performed using against SARS-CoV-2 S (1:1500, Sino Biological) and secondary Alexa Fluor 647 antibody (1:1000 dilution, #A-21246 ThermoFisher Scientific) and DAPI (0.2 µg/mL). Cells were imaged and analyzed using the Tecan Spark Cyto (Crailsheim, Germany). For immunohistochemical staining, the primary antibody was detected with a peroxidase-conjugated anti-rabbit secondary antibody (1:1,000, Dianova), followed by the addition of AEC substrate. The S positive area was scanned and quantified by the Bioreader 7000-F-Z-I microplate reader (Biosys, Karben, Germany). The results are expressed as a percentage of inhibition relative to the virus control, which received no drug.

### Viability assay

Cell viability was determined by 3-(4,5-dimethylthiazol-2-yl)-2,5-diphenyltetrazolium bromide (MTT) assay modified after Mosmann [52], as previously described [53]. Confluent cell cultures in 96-well plates were incubated with the ODN for 48 h. Then, 25 µL of MTT solution (2 mg/mL in PBS) was added per well, and the plates were incubated at 37 °C for an additional 4 h. After this, the cells were lysed using 200 µL of a buffer containing 20% (w/v) sodium dodecylsulfate and 50% (v/v) N,N-dimethylformamide with the pH adjusted to 4.7 at 37 °C for 4 h. Absorbance was determined at 570 nm for each well using a 96-well multiscanner (Tecan). After subtracting the background absorption, the results are expressed as percentage viability relative to the untreated control.

### Effect of GQ20-PTO on infected ALI cultures

ALI HBE cultures were infected with Omicron BA.1 (MOI 1) from the apical site for 2 h in the presence of GQ20-PTO (both apical/basal). Then the infection medium from the apical site was removed, and cells were washed three times with PBS. After 48 h post-SARS-CoV-2 infection, cells were collected for immunoblot analysis of virus replication and JAK/STAT signaling.

### One replication cycle assay

This assay was performed as previously described [54]. Briefly, to test (a) the effect of GQ20-PTO on virus entry, Calu-3 cells were infected with SARS-CoV-2 Omicron BA.1 at MOI 2 together with different concentrations of GQ20-PTO (5 or 1.25 µM). After 1 h at 37 °C, non-adherent viruses were washed away using PBS, and new medium was added. To test (b) post-entry effects GQ20-PTO was only added after the washing step. After incubation for 8 h cells, the cellular RNA was extracted and SARS-CoV-2 sgRNA of E gene was analyzed by quantitative RT-PCR. Briefly, intracellular RNA isolation was carried out using the RNeasy 96QIAcube HT Kit (Qiagen) according to the manufacturer’s protocol. Detection of selected targets was performed with Luna Universal One-Step RT-qPCR (New England BioLabs, Frankfurt, Germany) according to the manufacturer’s protocol using the following primers: TBP (fw: 5′-ATCAGAACAACAGCCTGCC-3′; rev: 5′-GGTCAGTCCAGTGCCATAAG-3′); SARS-CoV-2 E gene (fw: 5′-ACAGGTACGTTAATAGTTAATAGCGT-3′; rev: 5′-ATATTGCAGCAGTACGCACACA-3′). Using the 2^−ΔΔ*C*t^ method, E gene RNA levels at 8 h post infection were normalized to TBP (TATA binding protein) to account for differences in RNA loading and then normalized to the level of E gene expression quantified in the same way after 2 h post infection.

### Pseudotyped virus infection assay

Cell-free viral particles-containing supernatants were generated by transient transfection of 293 T cells with four plasmids using the calcium phosphate transfection method as described previously [24]. Protocols are available at the LeGO website (http://www.LentiGO-Vectors.de). 10 µL/well of a luciferase expressing lentiviral vector pseudotyped with Omicron BA.1 or BA.5 spike protein of VSV-G control were added to confluent Calu-3 cells in a 96-well plate and incubated in the presence of GQ20-PTO for 48 h at 37 °C. Then luciferase activity was measured by adding 10 µL of Steady-Glo Luciferase Detection Reagent (Promega, Madison, WI, USA) per well, and the microplate was incubated in the dark for 15 min. Luminescence was measured using GloMax Multi Detection System (Promega).

### NSP13 expression in HEK293T cells

SARS-CoV-2 NSP13 was expressed in HEK293T cells using the lentiviral vector pLVX-EF1alpha-SARS-CoV-2-nsp13-2xStrep-IRES-Puro (Addgene plasmid #141379). Packaging of the vector was done as described [55], using the second-generation packaging plasmid psPAX2 (Addgene #12260). Stably transduced cells expressing NSP13 were selected using puromycin, expanded, and treated with 4 µM GQ20-PTO for 24 h. Consequently, protein extracts were subjected to western blot and analyzed for expression of NSP13.

### SARS-CoV-2 NSP13 helicase assay

The helicase assay was performed as described [56]. Briefly, a dimer of fluorescent-labeled Cy3-5´-GTCACTGTTCGAGCACCA-3´ and quencher-labeled BHQ-5´-CGCAGTCTTCTCCTGGTGCTCGAACAGTGAC-3 (complementary sequences are underlined) was generated by heating (95 °C, 5 min) followed by cooling to room temperature. This probe served as a substrate for the SARS-CoV-2 helicase NSP13. The SARS-CoV-2 helicase activity assay was performed by mixing 100 nM substrate (dimer) with 100 nM NSP13 (Cayman Chemical, Tallinn, Estonia), 75 mM ATP, 2 µM capture probe (unlabeled 5´-GTCACTGTTCGAGCACCA-3´ used as trap), and 0.2, 1 and 4 µM GQ20-PTO or GQ20-PDE and consecutive incubation at 30 °C for 10 min. Helicase activity separates the Cy3-labeled ODN from the quencher-labeled complementary strand; an excess amount of capture probe avoids reannealing. After termination of the reaction by the addition of an equal volume of loading buffer (100 mM EDTA, 0.2% SDS, and 20% glycerol), the samples were separated by non-denaturating PAGE. Fluorescence, as a marker for helicase activity, was captured by using the LI-COR Odyssey Gel documentation system (Bad Homburg, Germany).

### SARS-CoV-2 NSP13 ATPase activity assay

The activity of the SARS-CoV-2 NSP13 ATPase was measured as described [8]. The ATPase assay is a colorimetric assay which makes use of the complexation of ATP-released phosphate by malachite green and molybdate (AM/MG reagent). In a first step, the following compounds were added to the reaction buffer (25 mM HEPES [pH 7.5], 50 mM NaCl, 5 mM MgCl_2_, 1 mM DTT): 0.25 mM ATP, 150 nM SARS-CoV-2 helicase NSP13 (Cayman Chemical, Tallinn, Estonia) and 4 µM GQ20-PTO or GQ20-PDE. As positive control served 1 mM Lumacaftor (Hycultec, Beutelsbach, Germany) as described [8]. After incubation at 37 °C for 20 min the AM/AG dye solution (Malachit Green Phosphate Assay Kit, Sigma-Aldrich) was added to the reaction buffer. After 5 min at room temperature, the production of phosphate was measured by monitoring the absorbance at 620 nm using a scanning multiwell spectrophotometer (ELISA-Reader ASYS Expert 96, Deelux Labortechnik, Gödenstorf, Germany).

### Temperature stability assay

NSP13 unfolding in the presence or absence of GQ20-PTO/PDE was evaluated by monitoring the fluorescence of the fluorophore SYPRO Orange (Invitrogen, Dreieich, Germany) using a real-time PCR device (QuantStudio 5, Thermo Fisher Scientific, Dreieich, Germany) according to the protocol described [57]. Briefly, 50 ng NSP13 protein (Cayman Chemical, Ann Arbor, Michigan, USA) was diluted in 10 mM HEPES (pH 7.5) buffer containing 200 mM NaCl in a total volume of 10 µL. The reactions were performed in 96-well PCR microtiter plates in the presence of 1/1000 SYPRO Orange. The temperature gradient was performed in steps of 0.3 °C per second, ranging from 25 to 95 °C. Tm values were calculated by using GraphPad Prism, fitting the curves to a Boltzmann sigmoidal equation, with all *R*^2^ > 0.998.

### Effect of GQ20-PTO/PDE on intracellular signaling

Calu-3 cells and human pulmonary fibroblasts were pre-treated with 4 µM GQ20-PTO and GQ20-PDE for 1 h followed by addition of 20 ng/mL IFNβ, 20 ng/mL IL-6, 10 ng/mL TNFα (all from Peprotech, Hamburg Germany) or 1 µM baricitinib (Hycultec, Beutelsbach, Germany). After 10 min cells were lysed in Strawn Buffer (20 mM HEPES [pH 7.5], 150 mM NaCl, 0.2% TritonX 100, 10% glycerol) supplemented with a protease inhibitor cocktail (Roche, Mannheim, Germany) and analyzed by immunoblot assay.

### Immunoblot analysis

The cellular lysates were sonicated and boiled for 5 min and separated on SDS-polyacrylamide gels. Consequently, proteins were immunoblotted to a PVDF membrane. The membranes were blocked in blocking buffer (TBS [pH 7.6], 0.1% Tween-20, 5% nonfat dry milk) for at least 3 h at 4 °C and then incubated with the following primary antibody: p-JAK1 (Tyr1022/1023, #3331S, 1:1000), JAK1 (#3344 1:1000), p-JAK2 (Tyr1008‚ #8082S, 1:1000), JAK2 (#3230, 1:1000), p-TYK2 (Tyr1054/1055, #68790, 1:1000), TYK2 (#14193, 1:1000), p-STAT1 (Tyr701, #9171, 1:1000), STAT1 (#14994, 1:1000), p-STAT2 (Tyr690‚ #88410, 1:1000), STAT2 (#72604, 1:1000), p-STAT3 (Tyr705, #9145S, 1:500), STAT3 (#9139, 1:1000) and beta-actin (#3700, 1:2000) (all from CST, Frankfurt, Germany). HBE ALI lysates were analyzed with primary antibodies against GAPDH (#2118, 1:4000, CST), ISG15 (#sc-166755,1:200, Santa Cruz Biotechnology), Mx1 (#37849, 1:1000, CST), SARS-CoV-2 Nucleocapsid (#40143-R019, 1:10 000, Sino Biological), STAT1 (#9172, 1:1000, CST), phospho-STAT1 Y701 (#9171, 1:1000, CST). For detection of NSP13 from NSP13-transduced HEK293T anti-NSP13 (#286909, 1:1000, Abcam) was used. Bound primary antibodies were detected by using rabbit anti-goat IgG-horseradish peroxidase conjugates (Dako, Frankfurt, Germany), visualized with the LumiGlo detection system (CST, Frankfurt, Germany), and captured with the LI-COR Odyssey Gel documentation system. Densitometric analysis of immunoblots was performed using ImageJ [58].

### Caspase 3/7 activity

Caspase 3/7 activity in infected ALI HBE was measured using the Caspase-Glo assay kit (Promega), according to the manufacturer’s instructions. Briefly, 100 µL of Caspase-Glo reagent was mixed with 100 μL of basal medium from ALI cultures and incubated at room temperature for 30 min. Luminescence intensity was measured using an Infinite M200 microplate reader (Tecan).

### Detection of G-quadruplexes (G4)

The formation of G4 was detected as described [23, 59]. Briefly, 0.2 µg 5′-Cy5-labeled ODN were mixed with 200 and 400 ng of the G4-specific antibody BG4 (Biozol ABA-AB00174-1.1, Eching, Germany) for 15 min at room temperature. After separation by 10% non-denaturing PAGE (100 V, corresponding to 14.7 V/cm) with 0.5× TBE fluorescence was captured using the LI-COR Odyssey Gel documentation system (Bad Homburg, Germany).

### Cellular uptake of ODN

The cellular uptake was tested in 2D cultures using Calu-3 cells and in 3D cultures using ALI HBE cultures (see above). After an incubation with 1 µM Cy3-labeled GQ20-PTO or GQ20-PDE for 2 h or 24 h stained with Hoechst 33324 to display nuclei and consecutively examined using the Operetta CLS High-Content Analysis System (Revvity, Hamburg, Germany).

### IFNα/β and IL-6

HEK-Blue™ IFNα/β and IL-6 (HEK293 reporter cells engineered to express human IFNAR1/R2 or IL-6) were purchased from Invivogen (Toulouse, France). These cells serve to study the activation of the IFNα/β or IL-6 signaling pathway by detecting the activation of an inducible SEAP (secreted embryonic alkaline phosphatase) reporter gene, which is under the control of (a) the IFN-α/β inducible ISG54 (IFN-stimulated gene 54) promoter or (b) STAT3, respectively. HEK-Blue cells seeded in 96-well plates at a density of 5 × 10^4^ cells/well were treated with 4 µM GQ20-PTO or GQ20-PDE for 24 h and then stimulated with either 0.5 × 10^4^ U/mL IFNβ or 1.5 ng/mL IL-6. After 24 h, the levels of SEAP activation are quantitatively determined by using the QUANTI-Blue assay (Invivogen). Briefly, 20 μL of cell-free supernatant was transferred into a new 96-well plate, mixed with 90 μL QUANTI-Blue reagent, and incubated at 37 °C for 15 min. The SEAP activity was detected by monitoring the absorbance at 620 nm using a scanning multiwell spectrophotometer (ELISA-Reader ASYS Expert 96, Deelux Labortechnik, Gödenstorf, Germany).

### Detection of ROS

The development of reactive oxygen species (ROS) in live cells was detected and quantified using the CellROX kit according to the manufacturer’s instructions (C10444, Invitrogen). Briefly, 16HBE14o cells were seeded in MTP (7.5 × 10^3^ cells/0.28 cm^2^) and treated with 4 µM GQ20-PTO or GQ20-PDE for 24 h. Then cells were incubated with 1 mM H_2_O_2_ with and without 200 µM ascorbic acid. After loading the cells for 30 min with 25 µM CellRox dye, fluorescence was measured and quantified using the Operetta CLS High-Content Analysis System.

### Statistical analysis

The results are expressed as the mean ± SD. The Student’s *t* test was used for comparing two groups. Three or more groups were compared by ANOVA. The exact statistical details of each experiment can be found in the figure legends. GraphPad Prism 9 was used to visualize data and to determine IC_50_ and CC_50_. Graphical depiction of experiments was done by BioRender.

## Supplementary information

Suppl Fig S02 video

Suppl Fig S1

Suppl Fig S3

Suppl Fig S4

Suppl Fig S5

Suppl Fig S6

Original Western Blots
