# Supplementary figures and images for: Discovery of synthetic G-quadruplex DNA as SARS-CoV-2 helicase inhibitor with antiviral, anti-inflammatory and antioxidative properties
Source: Cell Death Discov. 2026 Mar 18;12:159. doi: 10.1038/s41420-026-03006-0 (PMC13039202; doi:10.1038/s41420-026-03006-0)

## Slide 1
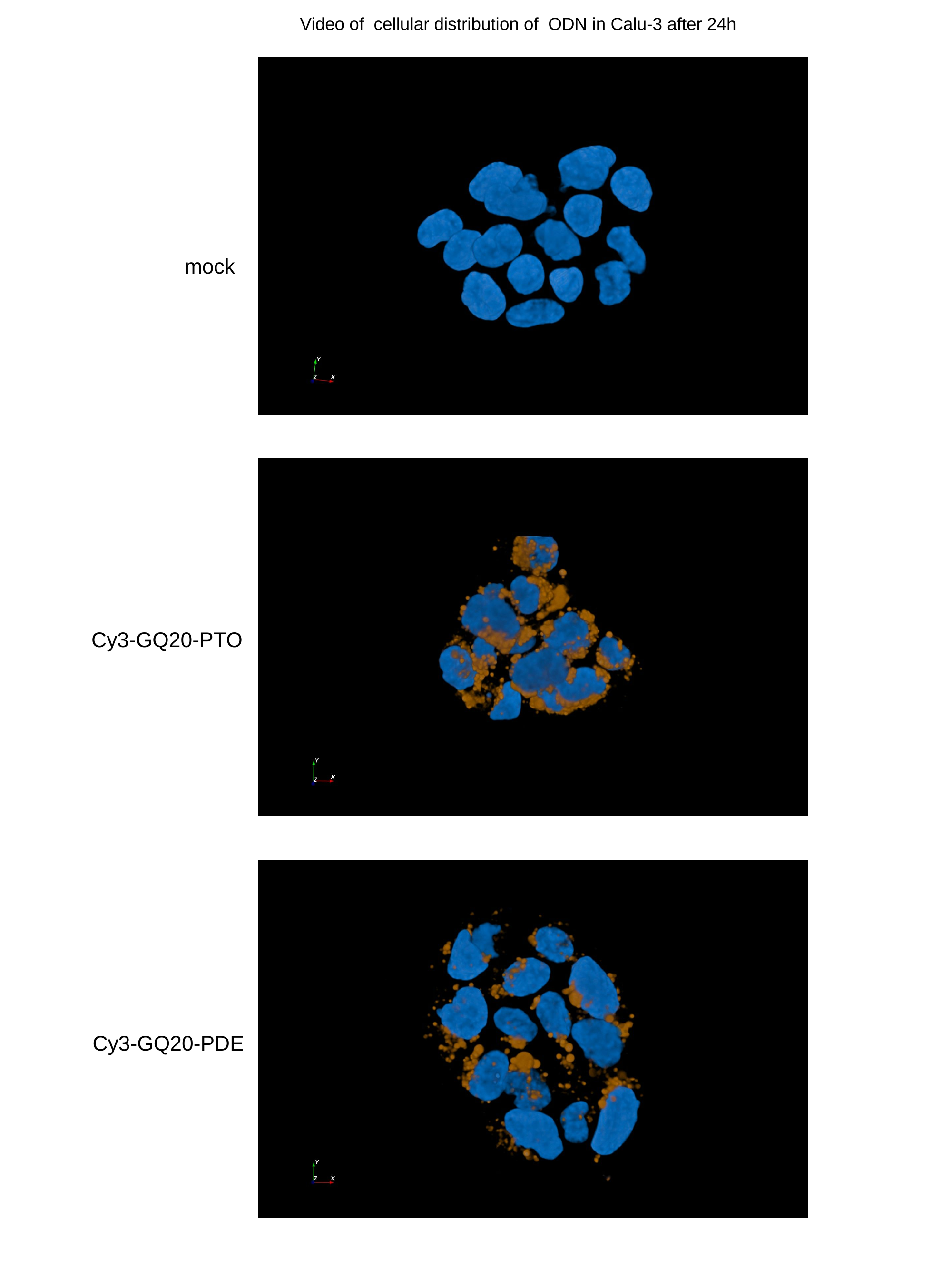

Video of cellular distribution of ODN in Calu-3 after 24h
mock
Cy3-GQ20-PTO
Cy3-GQ20-PDE

Supplement: Supplementary file 1 — Suppl Fig S02 video [file 41420_2026_3006_MOESM1_ESM.pptx]

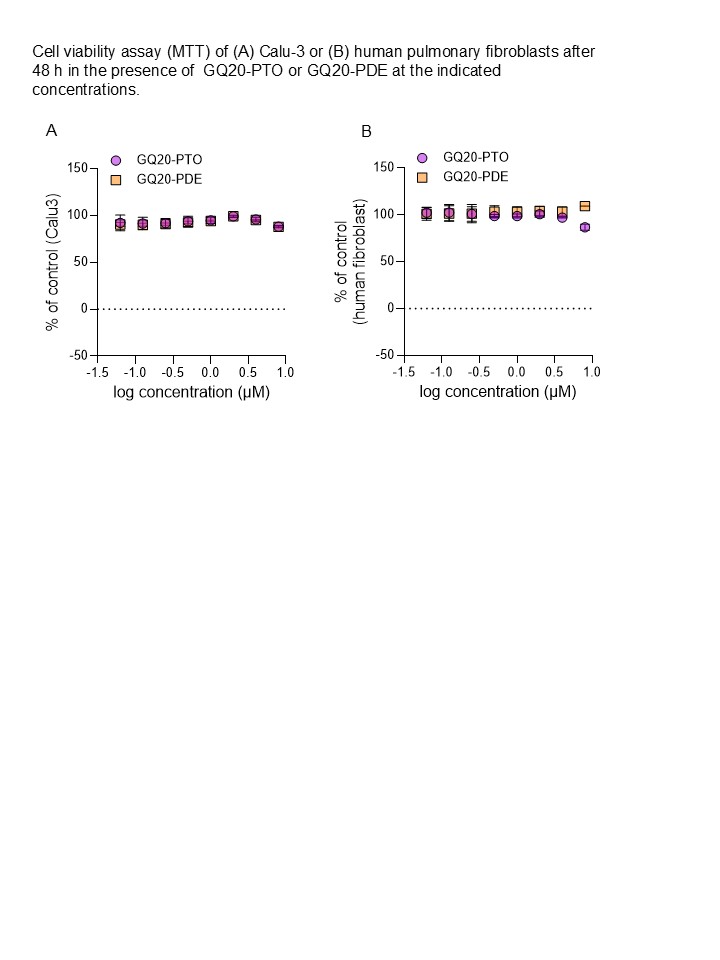

Supplement: Supplementary file 2 — Suppl Fig S1 [file 41420_2026_3006_MOESM2_ESM.jpg]

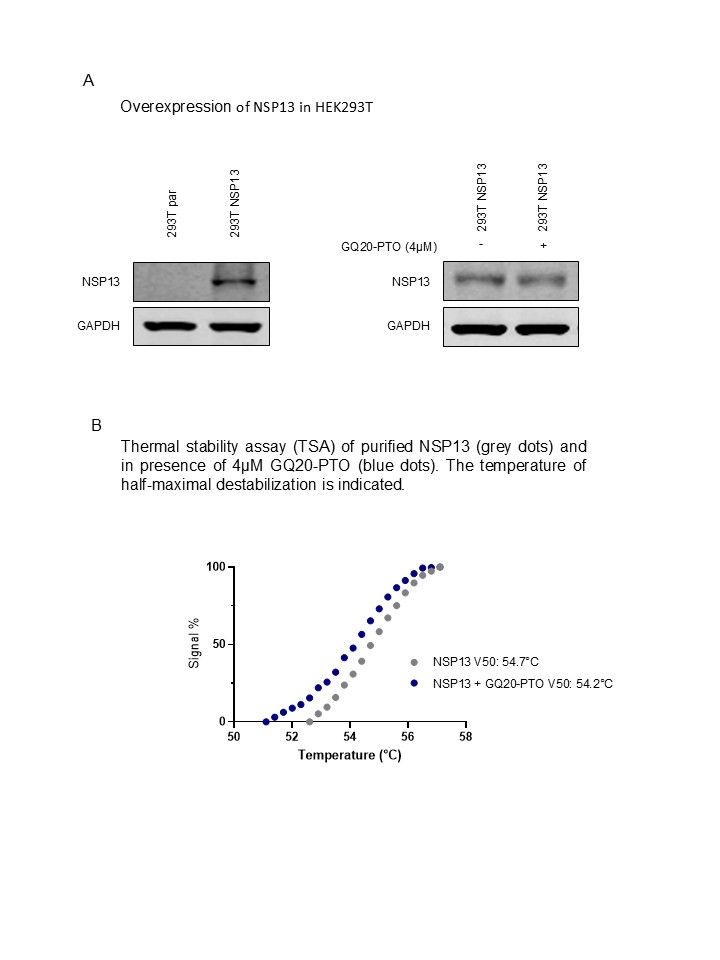

Supplement: Supplementary file 3 — Suppl Fig S3 [file 41420_2026_3006_MOESM3_ESM.jpg]

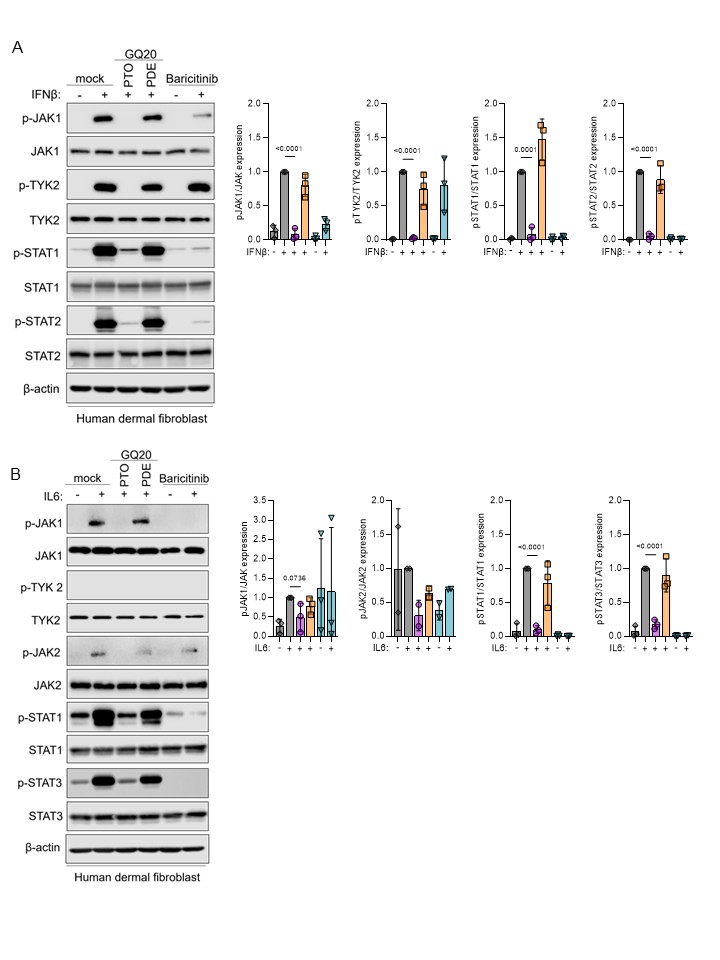

Supplement: Supplementary file 4 — Suppl Fig S4 [file 41420_2026_3006_MOESM4_ESM.jpg]

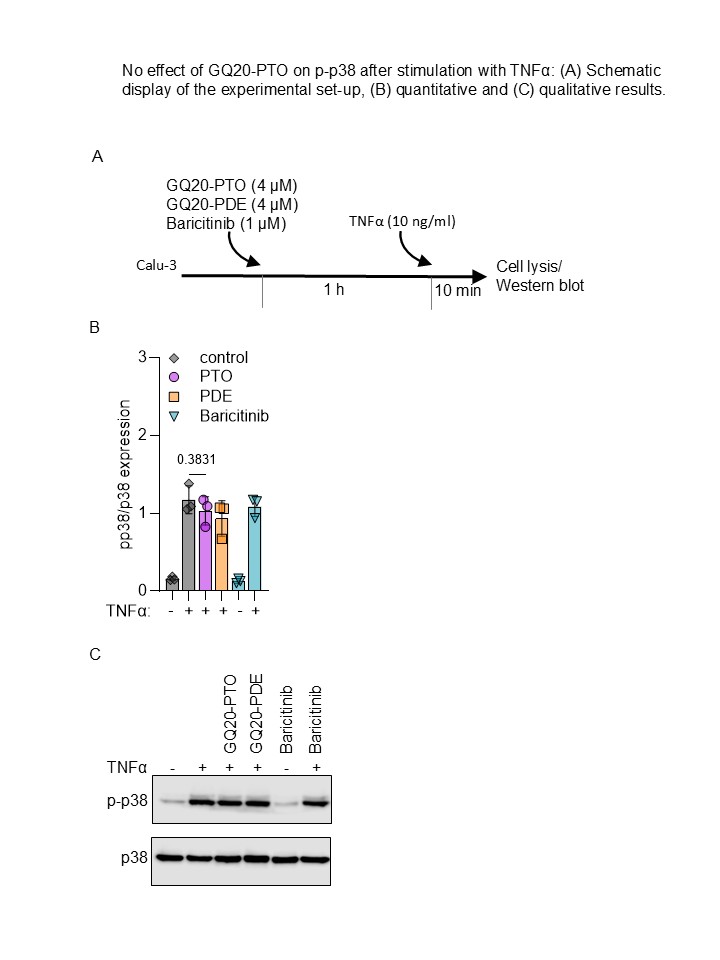

Supplement: Supplementary file 5 — Suppl Fig S5 [file 41420_2026_3006_MOESM5_ESM.jpg]

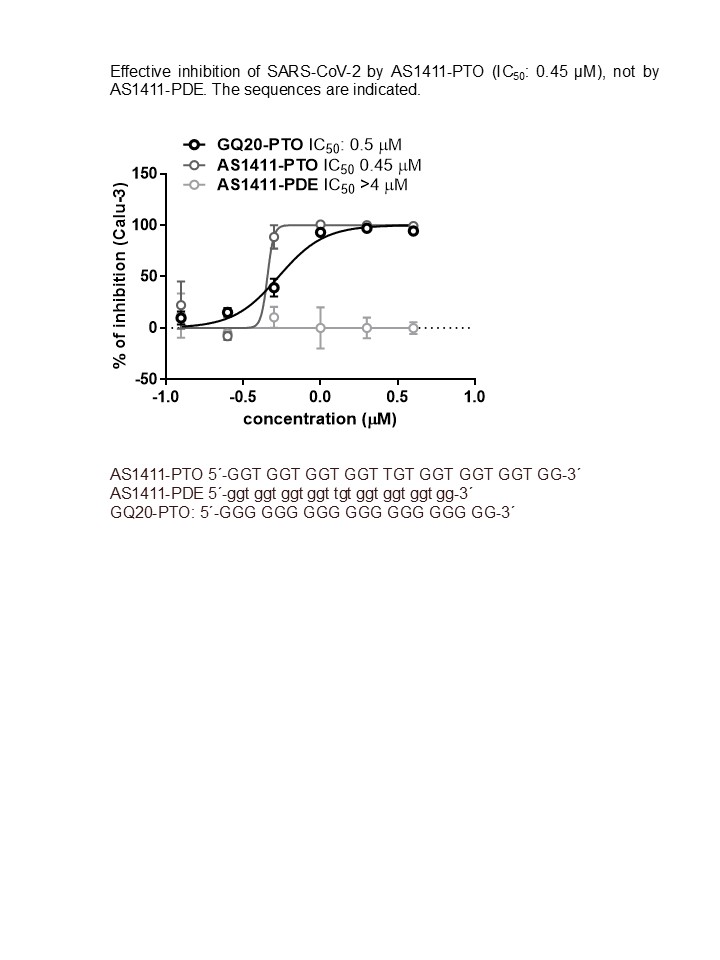

Supplement: Supplementary file 6 — Suppl Fig S6 [file 41420_2026_3006_MOESM6_ESM.jpg]
