# Supplementary material for: Discovery of synthetic G-quadruplex DNA as SARS-CoV-2 helicase inhibitor with antiviral, anti-inflammatory and antioxidative properties
Source: Cell Death Discov. 2026 Mar 18;12:159. doi: 10.1038/s41420-026-03006-0 (PMC13039202; doi:10.1038/s41420-026-03006-0)
Supplement: Supplementary file 7 — Original Western Blots [file 41420_2026_3006_MOESM7_ESM.pptx]

## Slide 1
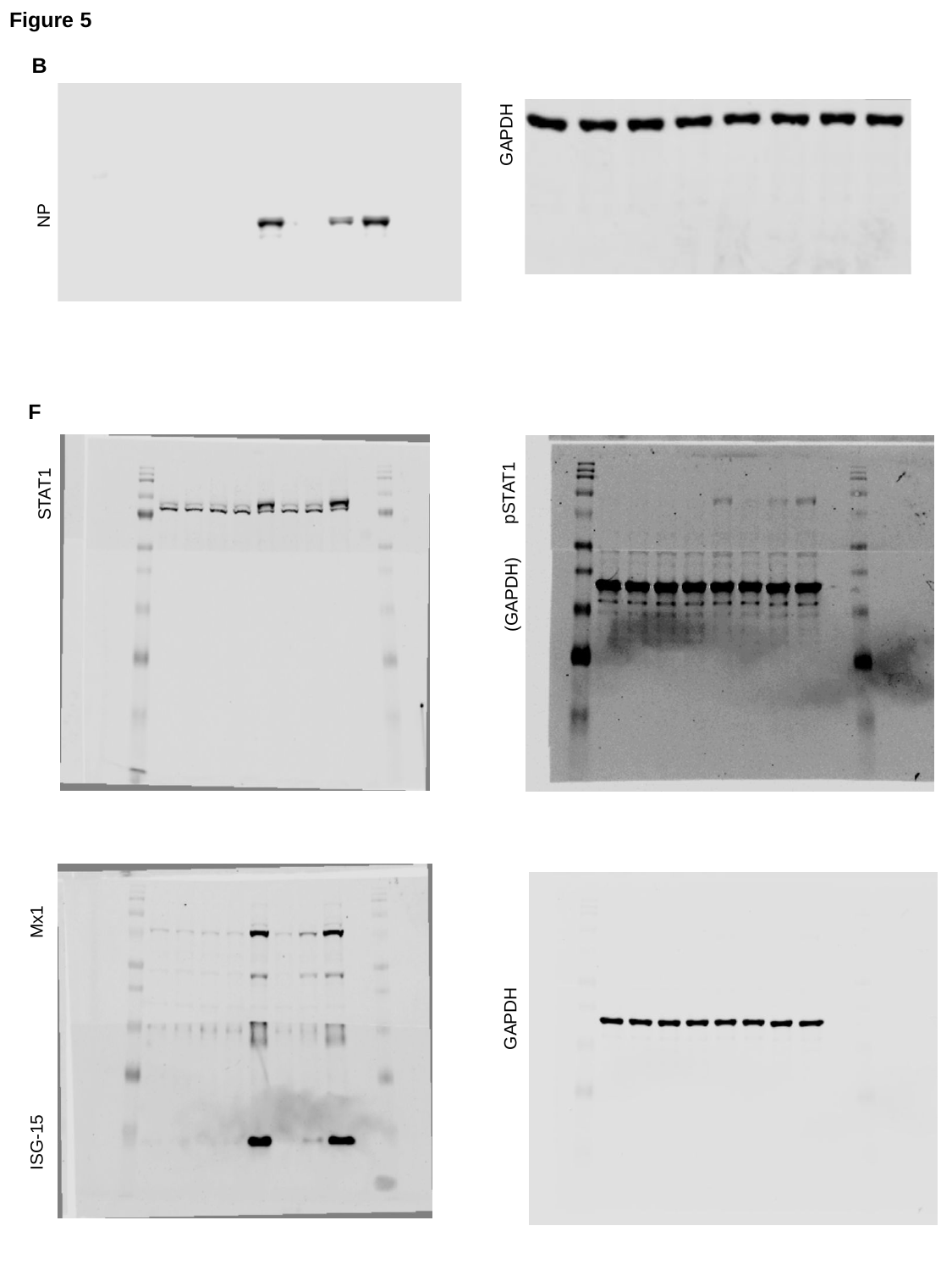

Figure 5
B
GAPDH
NP
F
STAT1
pSTAT1
(GAPDH)
Mx1
GAPDH
ISG-15

## Slide 2
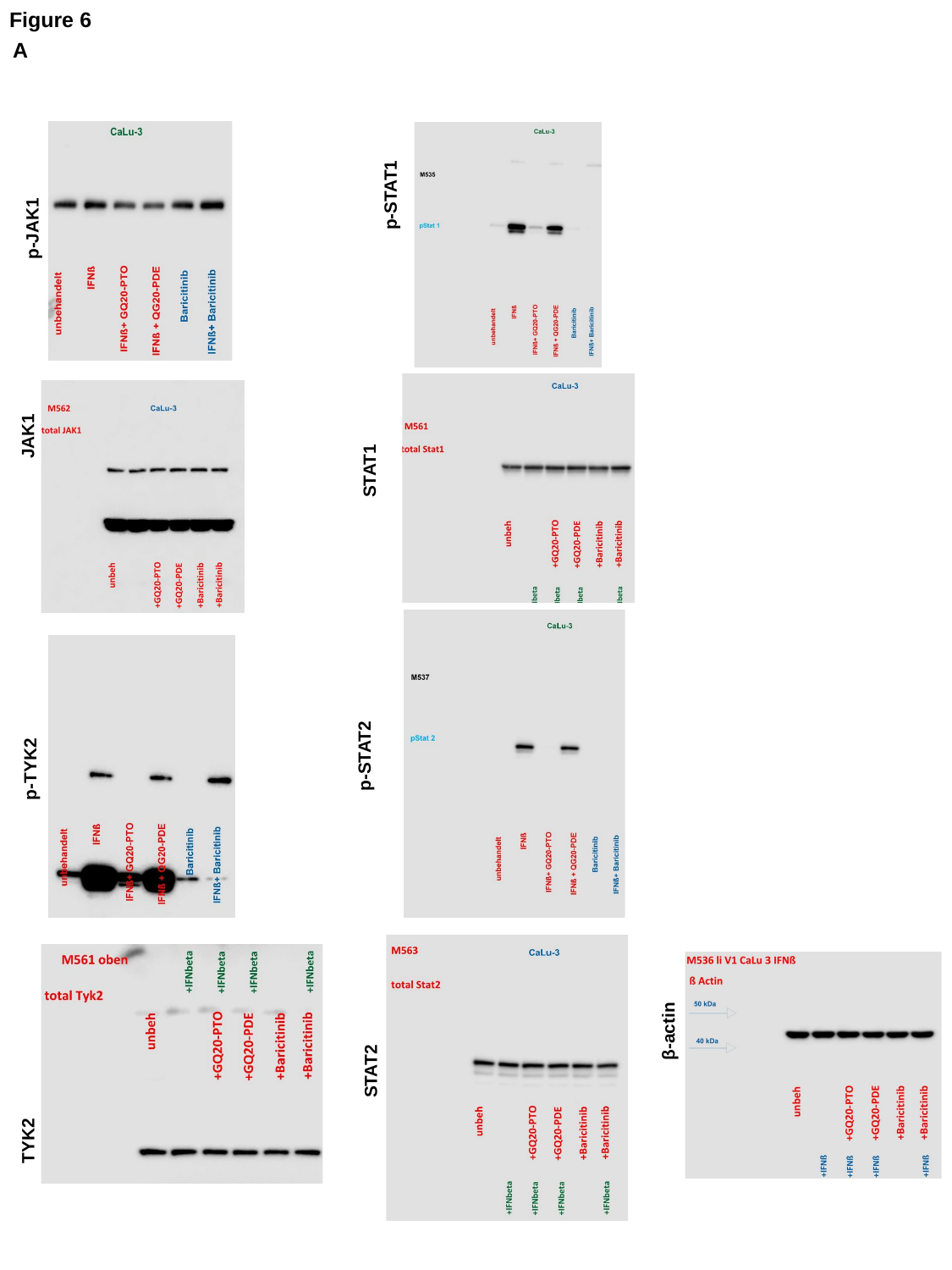

Figure 6
IFNβ
IFNβ
A
p-STAT1
p-JAK1
JAK1
STAT1
p-STAT2
p-TYK2
β-actin
STAT2
TYK2

## Slide 3
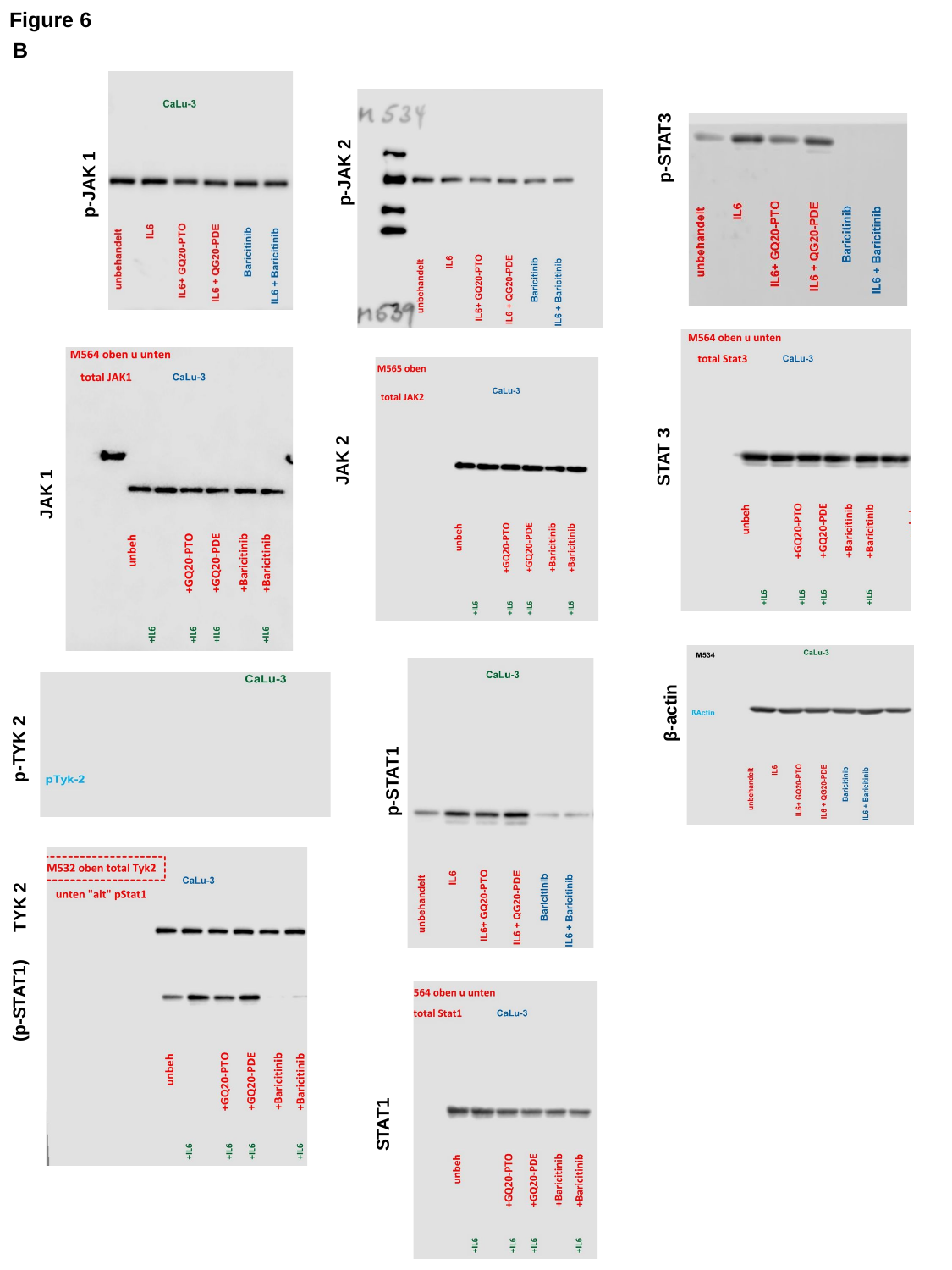

Figure 6
B
p-STAT3
p-JAK 2
p-JAK 1
JAK 2
STAT 3
JAK 1
β-actin
p-TYK 2
p-STAT1
TYK 2
(p-STAT1)
STAT1

## Slide 4
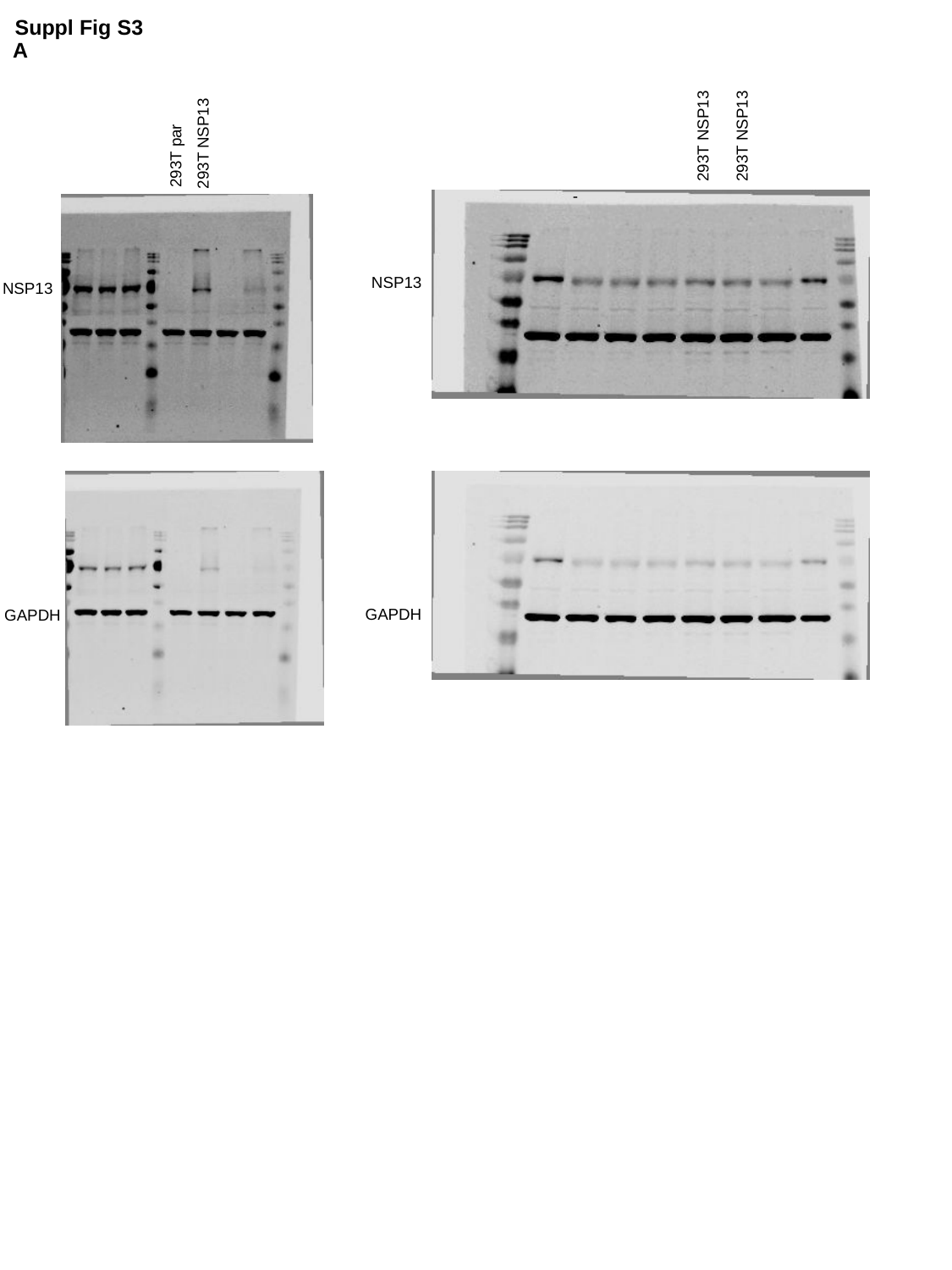

Suppl Fig S3
A
293T NSP13
293T par
293T NSP13
293T NSP13
-
+
NSP13
NSP13
+
GAPDH
GAPDH

## Slide 5
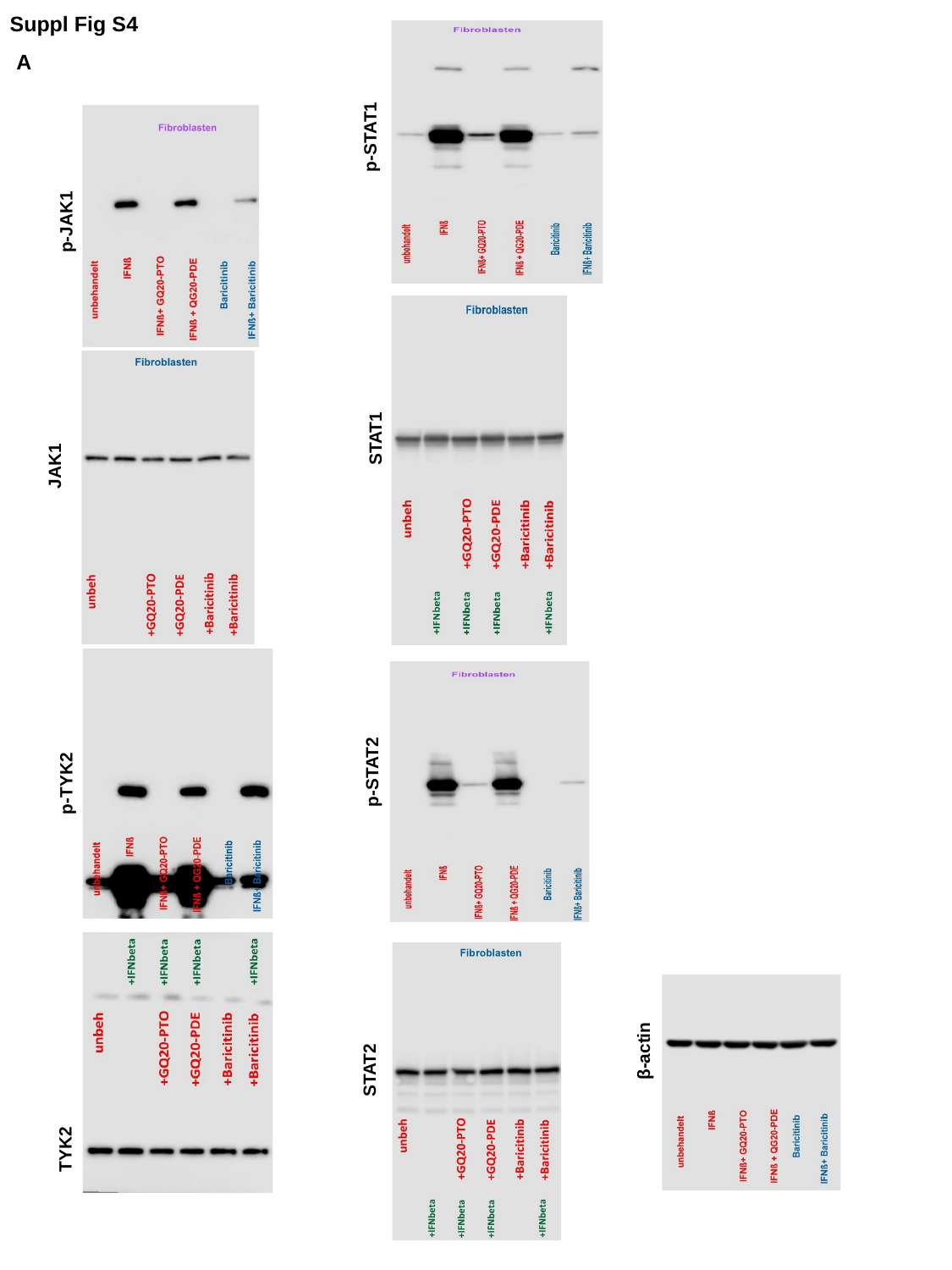

Suppl Fig S4
A
p-STAT1
p-JAK1
STAT1
JAK1
p-STAT2
p-TYK2
β-actin
STAT2
TYK2

## Slide 6
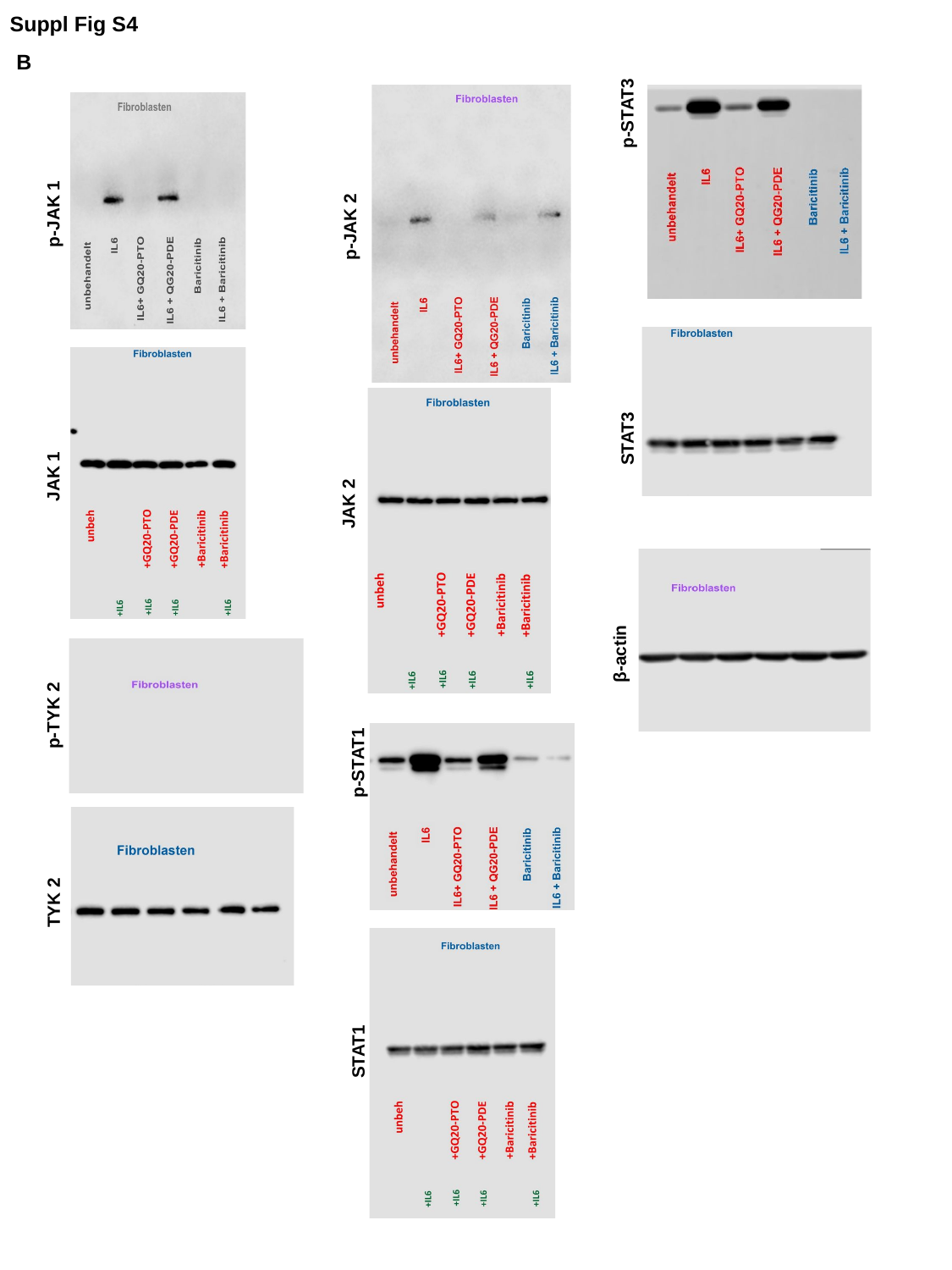

Suppl Fig S4
B
p-STAT3
p-JAK 1
p-JAK 2
STAT3
JAK 1
JAK 2
β-actin
p-TYK 2
p-STAT1
TYK 2
STAT1

## Slide 7
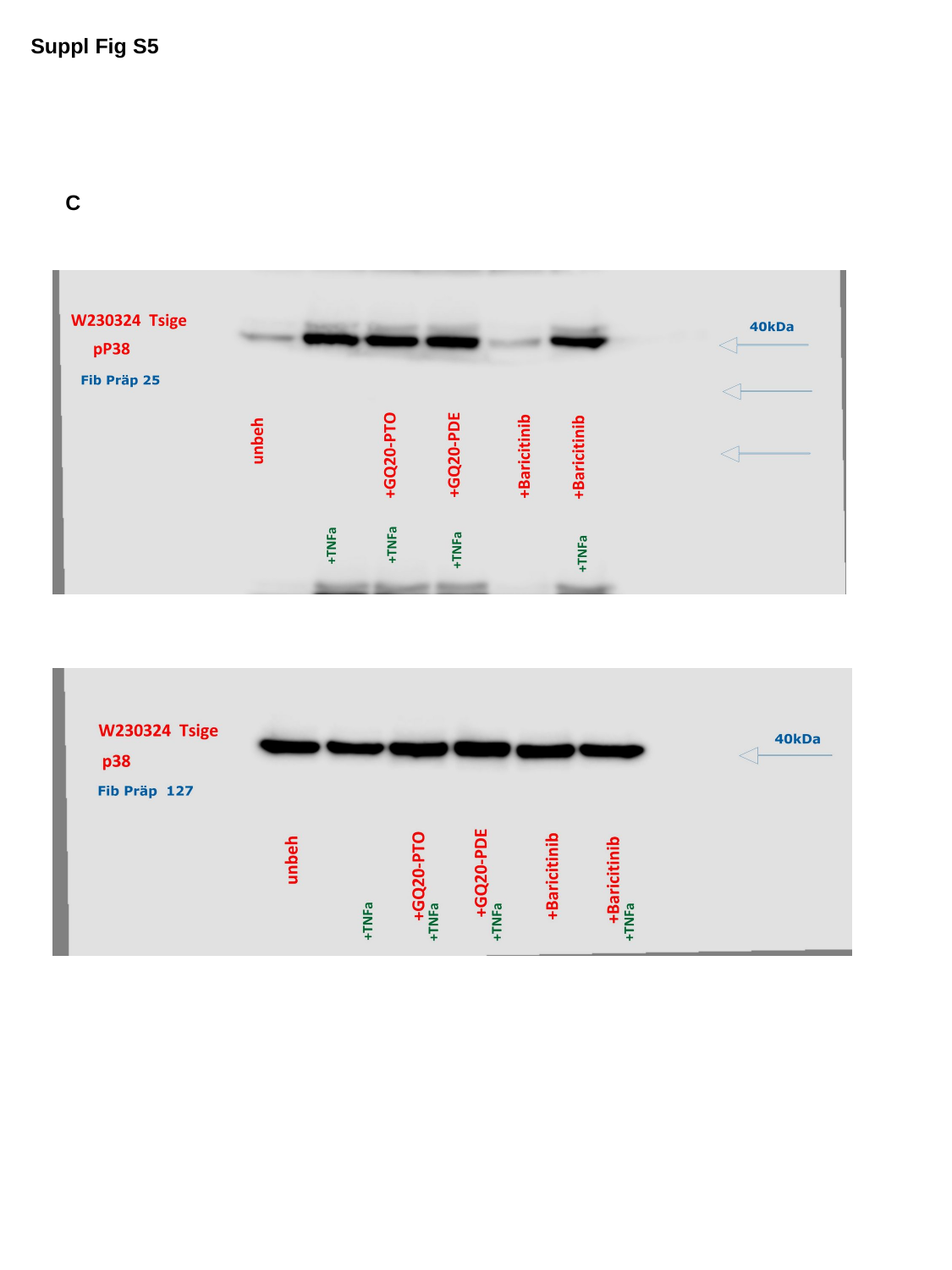

Suppl Fig S5
C
